# Signatures of CD4^+^ T and B cells are associated with distinct stages of chronic chagasic cardiomyopathy

**DOI:** 10.3389/fimmu.2024.1385850

**Published:** 2024-04-25

**Authors:** Isabela Natália Pascoal Campos do Vale, Gregório Guilherme Almeida, Inga Rimkute, Thomas Liechti, Fernanda Fortes de Araújo, Luara Isabela dos Santos, Priscilla Miranda Henriques, Manoel Otávio da Costa Rocha, Silvana Maria Elói-Santos, Olindo Assis Martins−Filho, Mario Roederer, Alan Sher, Dragana Jankovic, Andréa Teixeira−Carvalho, Lis Ribeiro do Valle Antonelli

**Affiliations:** ^1^ Biology and Immunology of Infectious and Parasitic Diseases Group, René Rachou Institute, Oswaldo Cruz Foundation-FIOCRUZ, Belo Horizonte, Brazil; ^2^ Integrated Research Group in Biomarkers, René Rachou Institute, Oswaldo Cruz Foundation-FIOCRUZ, Belo Horizonte, Brazil; ^3^ Vaccine Research Center, National Institute of Allergy and Infectious Diseases, National Institutes of Health, Bethesda, MD, United States; ^4^ Departament of Basic Science, Faculty of Medical Sciences of Minas Gerais, Belo Horizonte, Brazil; ^5^ Department of Clinical Medicine, Postgraduate Program in Infectious Diseases and Tropical Medicine, Federal University of Minas Gerais, Belo Horizonte, Brazil; ^6^ Department of Complementary Propedeutics, Faculty of Medicine, Federal University of Minas Gerais, Belo Horizonte, Brazil; ^7^ Immunobiology Section, Laboratory of Parasitic Diseases, National Institute of Allergy and Infectious Diseases, National Institutes of Health, Bethesda, MD, United States

**Keywords:** chagas disease, Trypanosoma cruzi, cardiomyopathy, CD4 + T cells, B cells, multifunctional, effector, memory

## Abstract

**Introduction:**

Chagas disease is a neglected parasitic disease caused by *Trypanosoma cruzi*. While most patients are asymptomatic, around 30% develop Chronic Chagasic Cardiomyopathy (CCC).

**Methods:**

Here, we employed high-dimensional flow cytometry to analyze CD4^+^ T and B cell compartments in patients during the chronic phase of Chagas disease, presenting the asymptomatic and mild or moderate/severe cardiac clinical forms.

**Results:**

Effector CD27^-^CD4^+^ T cells were expanded in both CCC groups, and only mild CCC patients showed higher frequencies of effector memory and T follicular helper (Tfh) cells than healthy donors (CTL) and asymptomatic patients. Unsupervised analysis confirmed these findings and further revealed the expansion of a specific subpopulation composed of Tfh, transitional, and central memory CD4^+^ T cells bearing a phenotype associated with strong activation, differentiation, and exhaustion in patients with mild but not moderate/severe CCC. In contrast, patients with mild and moderate/severe CCC had lower frequencies of CD4^+^ T cells expressing lower levels of activation markers, suggesting resting status, than CTL. Regarding the B cell compartment, no alterations were found in naïve CD21^-^, memory cells expressing IgM or IgD, marginal zone, and plasma cells in patients with Chagas disease. However, expansion of class-switched activated and atypical memory B cells was observed in all clinical forms, and more substantially in mild CCC patients.

**Discussion:**

Taken together, our results showed that *T. cruzi* infection triggers changes in CD4^+^ T and B cell compartments that are more pronounced in the mild CCC clinical form, suggesting an orchestrated cellular communication during Chagas disease.

**Conclusion:**

Overall, these findings reinforce the heterogeneity and complexity of the immune response in patients with chronic Chagas disease and may provide new insights into disease pathology and potential markers to guide clinical decisions.

## Introduction

Chagas disease is a neglected tropical parasitic disease caused by *Trypanosoma cruzi* and still represents a major public health problem. It is estimated that 7 million individuals are infected worldwide, causing approximately 12 thousand deaths annually (1, 2). While most patients affected by *T. cruzi* are asymptomatic, about 30% of infected individuals develop Chronic Chagas Cardiomyopathy (CCC) (3, 4), which is recognized as the most critical clinical manifestation of the disease (5, 6). Patients with CCC exhibit electrocardiographic changes that can lead to severe arrhythmias, heart failure, and thromboembolic phenomena. Therefore, sudden death represents a constant risk at any stage of CCC (7).

The specific immunological mechanisms involved in establishing and persisting of the different clinical forms of Chagas disease are still unknown. Indeed, the immune response and the balance between the effector and regulatory mechanisms seem to determine the disease outcome (8). Clinical manifestations in CCC patients are attributed to an intense inflammatory infiltrate in the myocardium, composed mainly of CD8^+^ and CD4^+^ T cells, macrophages, and, to a lesser extent, B cells (9–11). CCC individuals present a Th1-type cellular response, mediated by high levels of IFN-γ and lower levels of modulatory cytokines, leading to an imbalanced regulation of the immune response, contributing to persistent pathology (12–16).

B cells play an essential role in the cellular immune response by activating and presenting antigens to T cells, influencing cytokine production and tissue inflammation, thereby linking innate and adaptive immunity (5, 17). Although B lymphocytes develop an effective immune response to *T. cruzi* in the initial stage (18, 19), the antibodies primarily produced against *T. cruzi* surface antigens may not completely resolve the infection, allowing the establishment of chronic disease (17). Therefore, an early and effective B cell response is essential for eliminating the parasites since *T. cruzi* reduces peripheral blood CD27^+^ B cells, and memory B cells affect the IgG-specific response (20). Furthermore, various B cell defects have been reported in *T. cruzi*-infected individuals, such as polyclonal B cell activation, hypergammaglobulinemia and secretion of non-specific antibodies and autoantibodies contributing to parasite persistence and pathology (21–24).

Development of germinal centers (GC) and class-switching of immunoglobulins are dependent on Tfh cells (25–27). Dysregulation of this cell subset has been reported in autoimmune diseases, cancer, and infections associated with abnormal B cell responses (28, 29). The contribution of Tfh cells to the abnormal B cell responses in *T. cruzi* infection is still poorly understood, but it has been suggested that Tfh cells dysregulation contributes to Chagas disease progression (20, 21, 30).

The complex immune response surrounding CD4^+^ T cell phenotypes and B cell activity during CCC suggests a significant persistent inflammation, while immunoregulatory mechanisms seem to be progressively restrained. Therefore, the association between circulating T helper cells and B cell subsets and CCC clinical form deserve further investigation. Notably, recent study established a positive correlation between immune molecules detected in the blood and in heart tissue in patients with Chagas disease (31). Hence, we created two high-dimensional flow cytometry panels to phenotype CD4^+^ T and B cells from patients with distinct clinical phases of chronic Chagas disease. Our supervised and unsupervised data analysis revealed immune signatures associated with the immunopathological development of CCC.

Our findings revealed distinct features based on the heterogeneity of effector and memory phenotypes in patients with chronic Chagas disease. Notably, an inflammatory environment associated with an exhaustion profile was marked by highly differentiated CD4^+^ T cells and B cells undergoing isotype switching. These changes were pronounced in symptomatic patients, particularly during mild CCC, suggesting their significant association with pathophysiological mechanisms of cardiac disease.

## Population, materials, and methods

### Study population

Adults (18 to 65 years old) with positive serology for Chagas disease in at least two distinct diagnostic methods, in accordance with the II Brazilian Consensus on Chagas disease (3), were included in this study at Alda Lima Falcão Outpatient Clinic at René Rachou Institute (IRR), Belo Horizonte, Minas Gerais, Brazil. Exclusion criteria comprised severe dilated cardiomyopathy, renal and/or liver failure, and comorbidities significantly impacting life expectancy.

Chagas disease patients were categorized by Chagas disease specialized physicians, according to the American Heart Association: Stage A (indeterminate, here denoted as asymptomatic form, n = 8) represents patients at risk for developing cardiac disease with no symptoms or altered electrocardiogram; Stage B1 (mild cardiomyopathy, n = 12) includes patients with electrocardiographic changes, but with normal global ventricular function and no current or previous heart failure symptoms; Stage B2/C/D (moderate/severe cardiomyopathy, n = 8) comprises patients with structural cardiomyopathy and global ventricular dysfunction and having or not heart failure symptoms. The control group (CTL) consisted of nine healthy donors from the National Institutes of Health blood bank, USA, and five from IRR, Brazil, testing negative for Chagas disease. Patients who were treated (32%) received benznidazole at 5 mg/Kg/day for 60 days according to the guidelines from Brazilian Consensus in Chagas Disease. Volunteers enrolled in different groups were similar in age and sex, but differences were found in both percentage of left ventricle ejection fraction, LVEF (%), and left ventricle diameter, LV (mm), between B1 versus B2-C-D. A concise overview of each patient’s metadata and demographics can be found in the (**Supplementary Table 1**).

### Ethics statement

The protocols were approved by the Ethical Committee on Human Research at René Rachou Institute, Oswaldo Cruz Foundation (CEP-IRR- CAAE: 95998418.8.0000.5091). Patients provided written informed consent before their inclusion in the research.

### PBMC isolation

Peripheral blood samples were collected in sodium heparin tubes, and after centrifugation at 1,000 x g, 10°C for 10 minutes, plasma samples were separated and stored at -80°C. Cells were reconstituted 1:1 (v/v) in RPMI 1640 (Sigma-Aldrich, St Louis, MO, USA) and the peripheral blood mononuclear cells (PBMC) were isolated by slowly transferring blood diluted in phosphate-buffered saline (PBS) (1:2) on top of Ficoll–Paque gradient (Sigma). Samples were centrifugated at 700 x g for 40 min without break. Subsequently, the PBMC layers were collected, washed, and cell concentrations and viability were determined using the Countess II FL automated cell counter (Invitrogen Thermo Fisher Scientific). PBMC were frozen in fetal bovine serum (FBS, Gibco) with 10% dimethyl sulfoxide (DMSO; SIGMA) at -80°C for 24 hours using a freezing container (Mr. Frosty™, Thermo Scientific) and then maintained in liquid nitrogen until use.

### Cell staining for immunophenotyping

PBMC were thawed using thawsome adapted into a 15 mL tube containing RPMI 1640, 10% FBS, and benzonase nuclease (20 U/ml; Novagen, MilliporeSigma, Burlington, MA, USA) (32). Cell suspensions were washed in PBS and incubated with viability dye, diluted 1:1,000, for 15 min (Live/dead viability dye, UV blue, Thermo Scientific). After an additional wash in FACS buffer (PBS containing 0.1% BSA and 2 mM sodium azide), PBMC were incubated with two distinct antibody panels for 30 min (33). The panel designed for assessing CD4^+^ T cells and their subsets were based on (OMIP 58) (34) with modifications: anti-CCR6 (BB515, clone 11a90), CD57 (BB630, clone NK-1), CD244 (BB660, clone 2-69), CD127 (BB700, clone HIL-7R-M21), CXCR5 (BB790, clone RF8B2), TCR Vγ9 (PE, clone B3), TCR Vδ2 (PE-CF594, clone B6), CD161 (PE-Cy5, clone DX12), HLA-DR (PE-Cy5.5, clone TU36), ICOS (PE-Cy7, clone ISA-3), CD45RA (Ax700, clone HI100), CCR5 (APC-Cy7, clone 2D7/CCR5), CCR7 (BUV395, clone 150503), CD16 (BUV496, clone 3G8), CD56 (BUV563, clone NCAM16.2), CD279 (BUV661, clone EH12.1), CD95 (BUV737, clone DX27), CD4 (BUV805, clone SK3), CD122 (BV421, clone Mik-B3), CD3 (BV510, clone UCHT1), CD8 (BV570, clone RPA-T8), CD158 (BV605, clone DX27), CD28 (BV650, clone 28.2), CXCR3 (BV711, clone 1C6/CXCR3), CD38 (BV750, clone HB7), CD27 (BV786, clone L128). B cell subsets were assessed based on (OMIP 51) (35): anti-CD71 (FITC, clone M-A712), CD141 (BB630, clone 1a4), CD123 (BB660, clone 7G3), CD16 (BB700, clone 3G8), IgD (BB790, clone IA6-2), CD32 (PE, clone FUN-2), CD40 (PE-Dazzle594, clone 5C3), CD85j (PE-Cy5, clone GHI/75), CD11c (PE-Cy5.5, clone BU15), CXCR3 (PE-Cy7, clone G025H7), IgA (APC, goat polyclonal), CD27 (APC-R700, clone M-T271), CD19 (APC-H7, clone SJ25C1), CD1c (BUV395, clone F10/21a3), CD21 (BUV496, clone B-ly4), TACI (BUV563, clone 1a1-K21-M22), HLA-DR (BUV661, clone G46-6), IgG (BUV737, clone G18-145), CD20 (BUV805, clone 2H7), IL-21R (BV421, clone 2G1-K12), CD14 (BV510, clone MphiP9), IgM (BV570, clone MHM-88), BAFFR (BV605, clone 11C1), CD10 (BV650, clone HI10a), CD23 (BV711, clone M-L233), CXCR5 (BV750, clone RF8B2).

### Statistical analysis

T and B cell frequencies were determined manually (supervised) within the parental population or using unsupervised approaches within CD4^+^ or CD19^+^ cells. Frequencies and mean fluorescence intensity were obtained using FlowJo (v10). Flow-Self-Organizing Map (SOM) (v2.1.10), Pheatmap (v1.0.12), and Rtsne (v0.15) packages in R (v4.1.2) were employed for unsupervised analyses. For FlowSOM training we defined 196 clusters for CD4+ T cell and 100 clusters for B cell. Those were clustered in 50 metaclusters or FSOM populations and their distribution was visualized as minimum spanning trees (MST) (36). Pheatmap package was employed to generate heatmaps with hierarchical clustering calculated using Manhattan or Euclidean distances. Dimensionality reduction was achieved through t-distributed Stochastic Neighbor Embedding (tSNE). In this process, tSNE was set to run 5,000 iterations with a learning rate (eta) of 10,000 and an exaggeration factor of 36 until 1,000 iterations, following which momentum was introduced. Cluster frequencies were exported for subsequent statistical analyses. The frequencies of cell populations within each clinical group were analyzed using GraphPad Prism (v.10.3). The Shapiro-Wilk test was employed to assess the parametric distribution of variables within each clinical group. Clinical groups were compared with the Kruskal-Wallis test, followed by the Dunn’s test for multiple comparisons with correction via the Bonferroni method. Data were represented using boxes and whiskers representing mean, maximum, and minimal values and symbols representing volunteers. Significant differences (p < 0.05) were represented by asterisks.

## Results

### Chagas disease alters circulating CD4^+^ T cell compartments

**Figure 1:**
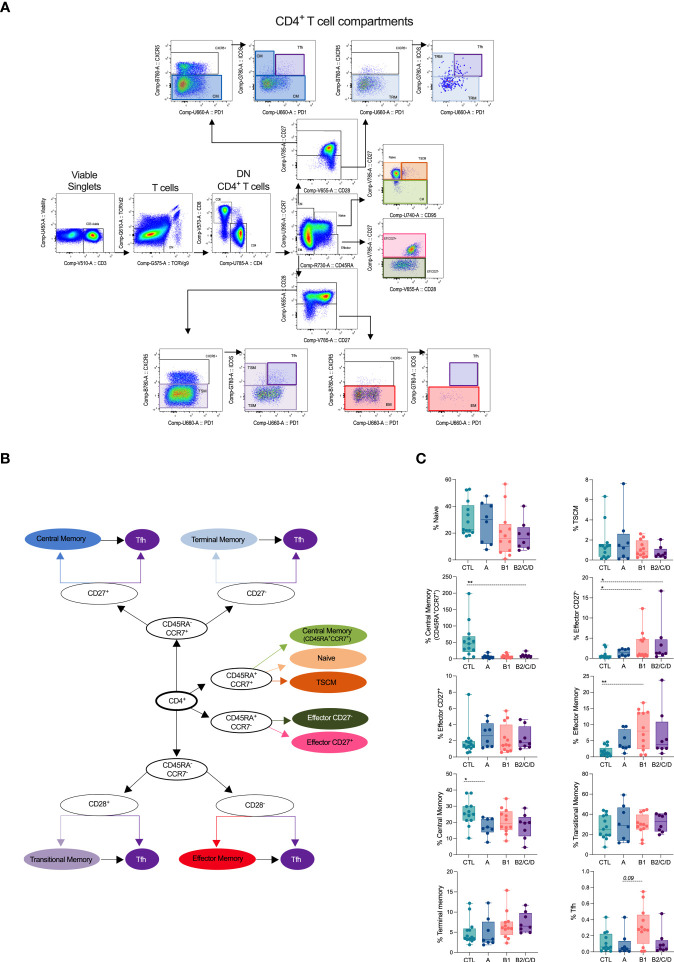
Chagas disease alters supervised defined CD4^+^ T cell compartments. **(A)** Flow cytometry gating strategy and organizational chart **(B)** to define CD4^+^ T subsets is shown. Viable cells expressing CD3, but lacking TCRVd2 and TCRVg9, were gated on CD4^+^ cells and naïve and memory subsets defined according to the expression of the CD45RA, CCR7, CD95, CD27 and CD28: central memory (CM, CD45RA^-^CCR7^+^CD27^+^), terminal memory (TRM, CD45RA^-^CCR7^+^CD27^-^), naïve (CD45RA^+^CCR7^+^CD27^+^CD95^-^), memory stem cell (TSCM, CD45RA^+^CCR7^+^CD27^+^CD95^+^), central memory (CD45RA^+^CCR7^+^CD27^-^), effector (CD45RA^+^CCR7^-^CD27^+^/CD27^-^), transitional memory (TSM, CD45RA^-^CCR7^-^CD28^+^), effector memory (EM, CD45RA^-^CCR7^-^CD28^-^). Tfh cells, defined by the co-expression of CXCR5, PD-1, and ICOS, were gated among each memory cell subset. **(C)** Percentage of CD4^+^ T cell subsets in healthy donors (CTL, n = 13), asymptomatic (A, n = 08), mild (B1, n = 12), and moderate/severe CCC (B2/C/D, n = 08), were defined by supervised analysis. Box and whiskers contain minimum and maximum values, median and interquartile range, and superimposed symbols represent individual values. Asterisks represent significant differences between the assigned groups. *p < 0.05, **p < 0.01.

CD4^+^ T cell subsets were manually defined within viable single CD3^+^ cells, lacking T cell receptors TCRVγ9 and TCRVδ2 (**Figures 1A, B**). We defined naïve and memory subsets based on the expression of CD45RA and CCR7. CD45RA^+^CCR7^+^ cells consisted of naïve (CD95^-^CD27^+^), stem cell-like memory (TSCM, CD95^+^CD27^+^), and CD27^-^ central memory cells (CM, CD27^-^). Effector cells (Eff) were defined as CD45RA^+^CCR7^-^, which we further distributed into CD27^-^ and CD27^+^ Eff. CD45RA^-^CCR7^+^ consisted of central memory cells and were further classified as CM (CD27^+^) and tissue-resident memory (TRM, CD27^-^). Lack of CD45RA, CCR7, CD28 and CXCR5 defined effector memory (EM), and of CD45RA, CCR7, CXCR5, but presence of CD28, were transitional memory cells (TSM). We identified Tfh cells based on the co-expression of CXCR5, PD-1, and ICOS within CM and EM subsets (**Figures 1A, B**).

CM cells were decreased in asymptomatic patients (A) compared to healthy donors (CTL). Patients with mild CCC (B1) displayed expansion of EM compared to CTL. Both mild and moderate/severe (B2/C/D) CCC patients showed increased frequencies of effector CD27^-^ cells. No differences were observed in the frequencies of naïve cells; however, patients with moderate/severe CCC showed a lower frequency of CD27^-^ CM among CD45^+^CCR7^+^ cells. Expansion of Tfh was observed in mild CCC compared to asymptomatic patients (**Figure 1C**).

### Patients with chagas disease show heterogeneity of CD4^+^ T cell subsets

**Figure 2:**
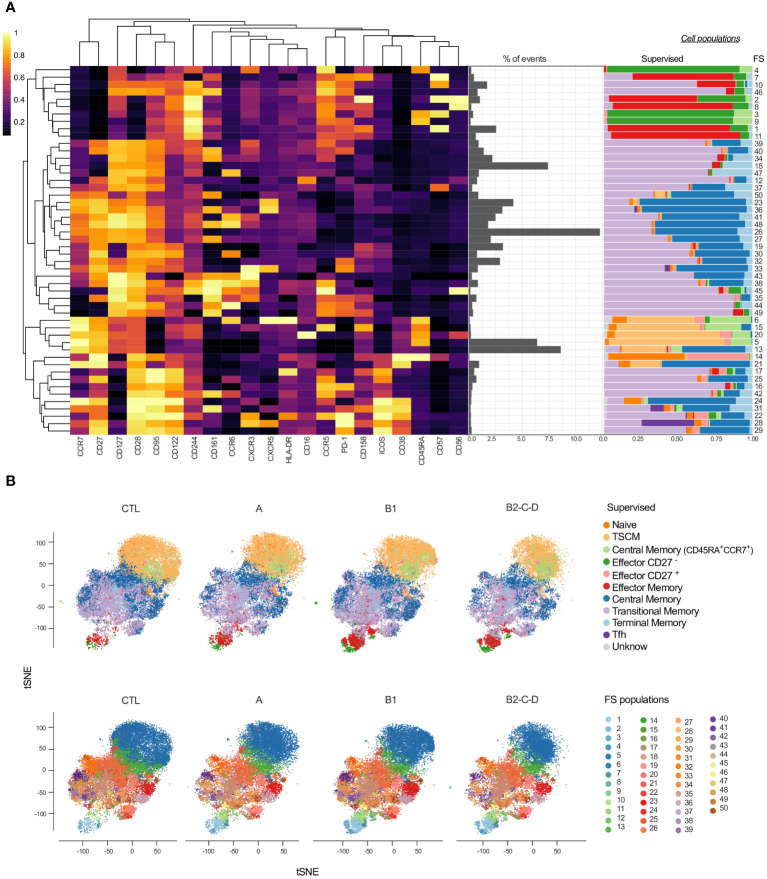
Overlay of CD4^+^ T cell subsets obtained by supervised and unsupervised analyses. **(A)** Heatmap depicts the expression of 21 surface antigens (x-axis), normalized by each marker, across 50 FS populations of CD4^+^ T cells. Markers are distributed by similar patterns based on hierarchical clustering. Proportion (middle panel) and composition of manually defined populations for each FS (right panel) are shown. **(B)** tSNE, normalized to represent the same number of events in each group, depicts manually/supervised (top panel) and unsupervised/FS (bottom panel) defined CD4^+^ T cell subsets. Healthy donors (CTL, n = 13), asymptomatic (A, n = 08), mild CCC (B1, n = 12), and moderate/severe CCC (B2/C/D, n = 08). Different colors are assigned for each supervised defined (left panel) and FS (right panel) subpopulations.

We applied unsupervised clustering using a self-organizing maps algorithm (FlowSOM - FS) to further explore distinct CD4^+^ T cell phenotypes among patients with different clinical forms of Chagas disease (**Figure 2**). A heatmap displays the heterogeneity of 50 clusters considering the entire study population. These clusters are composed of a distinct number of events (middle panel) and by distinct cell subsets according to the manual supervised gating (right panel) (**Figure 2A**). FS3, 4, and 9 consisted mainly of effector CD27^-^ and, to a lesser extent, of CM in naïve. FS7-8, FS1, and 11 comprise EM and a small proportion of effector CD27^-^. Most FS populations (17-29 and 39-49) are composed of TSM and/or CM cells. The majority of FS28 and 31 consisted of Tfh cells. FS6-21 contain cells expressing high levels of CCR7 and CD27, corresponding mainly to TSCM, some CM, and CM in CCR7^+^CD45RA^+^ cells. Effector CD27^+^ cells are a rare population concentrated in the FS14, along with naïve cells (**Figure 2A**).

We performed dimensionality reduction using tSNE and superimposed the data generated by supervised (**Figure 2B**, top panel) and unsupervised analyses (FS; **Figure 2B**, bottom panel) to evaluate qualitative differences in the cellular composition from patients with distinct clinical forms of Chagas disease. As shown in **Figure 1C**, we observed an increase in the frequency of effector CD27^-^, TRM, and EM cells with the progression of Chagas disease (groups A, B1, and B2/C/D) compared to CTL (**Figure 2B**, top panel). The tSNE generated with the FS populations revealed several other differences among the clinical forms (**Figure 2B**, bottom panel), including cells placed in the top area of the tSNE, where naïve subsets are present.

### Patients with chagas disease display an effector and activated profile of CD4^+^ T cells

**Figure 3:**
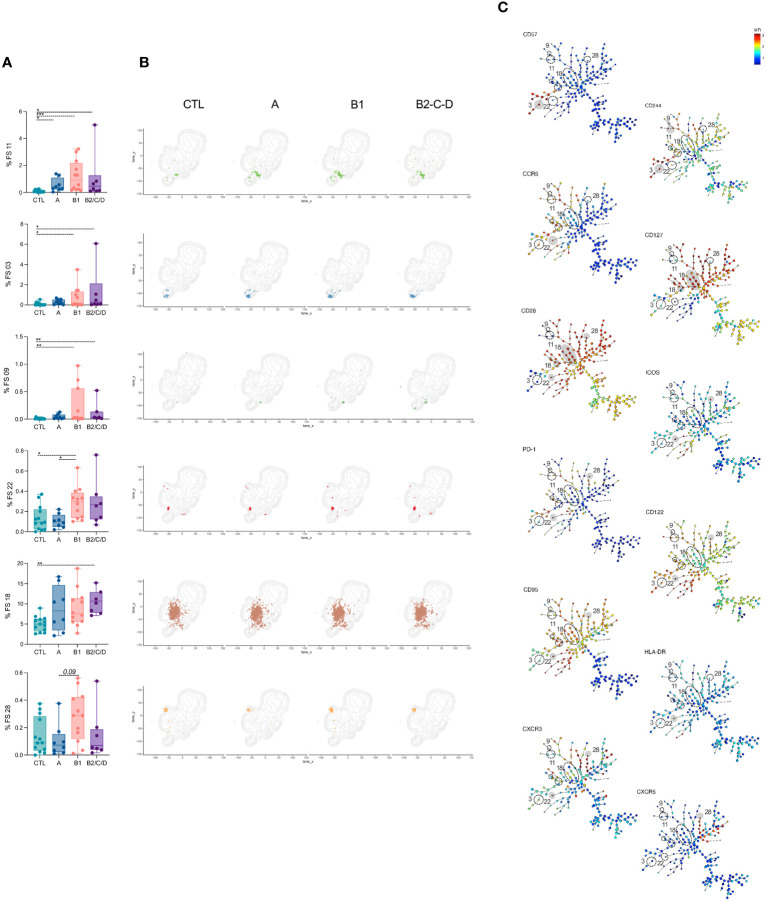
FS subpopulations of CD4^+^ T cells induced in patients with Chagas disease. **(A)** Percentage of FS11, 03, 09, 22, 18, and 28 within CD4^+^ T cells (from top to bottom) in healthy donors (CTL, n = 13), asymptomatic (A, n = 08), mild (B1, n = 12), and moderate/severe CCC (B2/C/D, n = 08). Box and whiskers contain minimum and maximum values, median and interquartile range, and superimposed symbols represent individual values. Asterisks represent significant differences between the assigned groups. *p < 0.05, **p < 0.01, ***p < 0.001. **(B)** tSNE contour plots show CD4^+^ T cells in gray and FS populations in colors, as in **Figure 2B**, from CTL and infected patients in different stages of Chagas disease. **(C)** Minimum spanning trees (MST), composed of 196 clusters, represent the distribution of 50 FS populations and show the differential expression, from lower (blue) to higher (red), of CD57, CD244, CCR5, CD127, CD28, ICOS, PD-1, CD122, CD95, HLA-DR, CXCR3, and CXCR5. FS populations **(A, B)** are delimited in dotted circles and highlighted in gray for higher expression.

**Figure 4:**
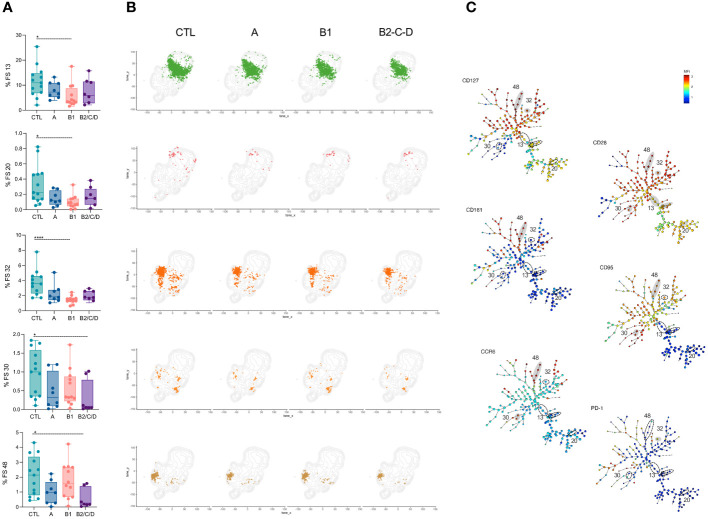
FS subpopulations of CD4^+^ T cells with central circulation and resting status are decreased in CCC patients. **(A)** Percentage of FS 13, 20, 32, 30, and 48 within CD4^+^ T cells (from top to bottom) in healthy donors (CTL, n = 13), asymptomatic (A, n = 08), mild (B1, n = 12), and moderate/severe CCC (B2/C/D, n = 08). Box and whiskers contain minimum and maximum values, median and interquartile range, and superimposed symbols represent individual values. Asterisks represent significant differences between the assigned groups. *p < 0.05, ****p < 0.0001. **(B)** tSNE contour plots show CD4^+^ T cells in gray and FS populations in colors, as in **Figure 2B**, from CTL and infected patients in different stages of Chagas disease. **(C)** Minimum spanning trees (MST), composed of 196 clusters, represent the distribution of 50 FS populations, and show the differential expression, from lower (blue) to higher (red), of CD127, CD28, CD161, CD95, CCR6, and PD-1. FS populations **(A, B)** are delimited in dotted circles and highlighted in gray for higher expression.

Among the 50 FS populations, 14 had frequencies altered in at least one of the three clinical forms compared to CTL (**Figures 3A**–**4A**). In addition, FS22 revealed different frequencies between asymptomatic and mild CCC patients (**Figures 3A, B**). In contrast, 35 clusters were similar among groups (**Supplementary Figure 1**). FS11 was composed almost exclusively of EM and was the only population increased in Chagas patients, independently of the clinical form, compared to CTL (**Figure 3A**). The frequencies of FS3 and 9, mainly composed of effector cells, were expanded in both mild and moderate/severe CCC patients compared to CTL. FS3, 9, and 11 have high expression of CD244, consistent with activation and effector functions, as depicted in the minimum spanning tree (MST) (**Figure 3C**, **Supplementary Figure 2**) (37, 38). FS3 also expresses high levels of CD57, a marker of highly activated cells (39, 40). The frequencies of FS22 were higher in mild CCC than in asymptomatic patients and CTL, while moderate/severe CCC showed a higher abundance of FS18 compared to CTL. Although clusters FS18 and 22 were composed mainly of transitional memory cells, they are essentially different; FS22 expresses a prominently activated phenotype characterized by the expression of high levels of PD-1, ICOS, CD28, CD95, CD122, CCR5, CXCR3, and HLA-DR, while FS18 expresses only high levels of CD28 and CD127 (**Figure 3C**). A higher frequency of FS28 was found in mild CCC compared to asymptomatic patients. FS28 is a heterogeneous population, composed of Tfh, transitional, and CM cells and characterized by high levels of CXCR5, PD-1, ICOS, and CD28 (**Figure 3C**).

**Figure 5:**
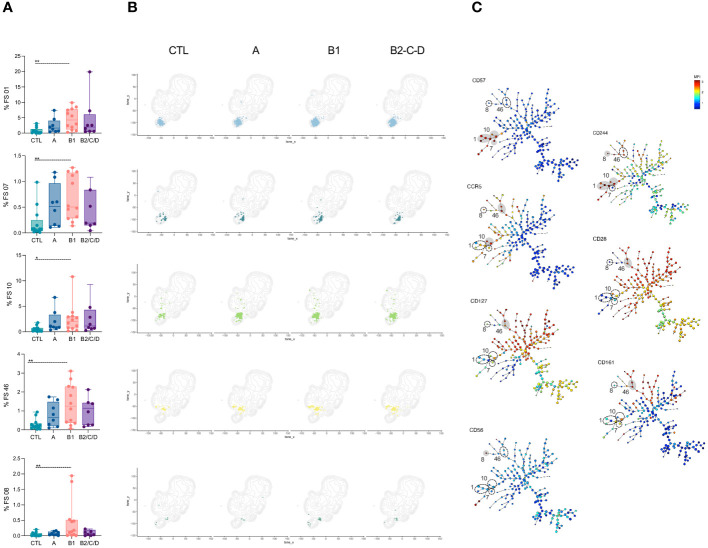
FS subpopulations of CD4^+^ T cells with memory phenotype are expanded in mild CCC patients. **(A)** Percentage of FS 01, 07, 10, 46, and 08 within CD4^+^ T cells (from top to bottom) in healthy donors (CTL, n = 13), asymptomatic (A, n = 08), mild (B1, n = 12), and moderate/severe CCC (B2/C/D, n = 08). Box and whiskers contain minimum and maximum values, median and interquartile range, and superimposed symbols represent individual values. Asterisks represent significant differences between the assigned groups. *p < 0.05, **p < 0.01. **(B)** tSNE contour plots show CD4^+^ T cells in gray and FS populations in colors, as in **Figure 2B**, from CTL and infected patients in different stages of Chagas disease. **(C)** Minimum spanning trees (MST), composed of 196 clusters, represent the distribution of 50 FS populations, and show the differential expression, from lower (blue) to higher (red), of CD57, CD244, CCR5, CD28, CD127, CD161, and CD56. FS populations **(A, B)** are delimited in dotted circles and highlighted in gray for higher expression.

Clusters FS1, 7, 8, 10, and 46 were expanded only in mild CCC patients compared to CTL (**Figures 5A, B**, top to bottom). FS01, 07, and 08 mainly composed of EM cells, while FS10 and FS46 mainly contained TM, EM, and effector CD27^-^ cells. Most FS with EM phenotype (FS1, 2, 7, 10) express high levels of CD57. FS01, 08, and 10 also express CD244, and FS08 expresses high levels of CD56. FS46 expresses high levels of CD161, which is associated with a memory phenotype (41), CCR5, CD127 and CD28 (**Figure 5C**).

A few FS populations had lower frequencies in CCC patients than in CTL (**Figure 4A, B**). Frequencies of FS13, FS20, and FS32 were lower in mild CCC than in CTL. FS13 was the most heterogeneous population, composed of CM, TSCM, and, to a lesser extent, of CM in CD45RA^+^CCR7^+^ and TSM cells. FS20 was mainly composed of cells with TSCM phenotype and FS32 of TSM and CM cells. These FS express very low activation markers, suggesting their central memory and resting status. Similarly, FS30 and FS48 were composed of TSM and CM cells, but their frequencies were lower in moderate/severe CCC than CTL. Both express high levels of CCR6, but F30 expresses high levels of CD95 and is less activated, while FS48 shows high levels of CD161, CD127, and CD28 (**Figure 4C**).

Taken together, CD4^+^ T cells from patients with Chagas disease bear an effector and activated phenotype that is pronounced in the mild CCC clinical form.

### Chagas disease alters circulating B cell compartments

**Figure 6:**
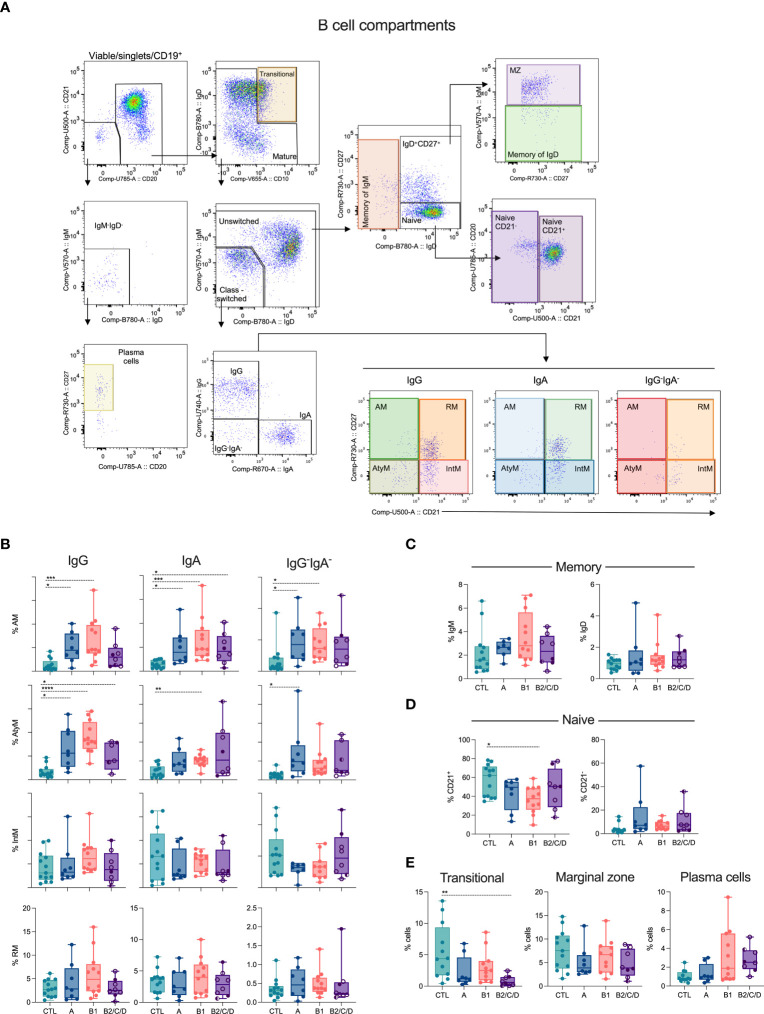
Class-switched memory subsets are expanded during Chagas disease. **(A)** Representative density plots showing B cell subsets. Viable cells expressing CD19 were defined as plasma cells (CD21^-^CD20^-^IgM^-^IgD^-^CD27^+^) or B cells (CD21^+^CD20^+^). B cells and their subsets were classified according to the expression of CD10, IgD, IgM, IgG, IgA, CD27 and CD21: transitional (IgD^+^CD10^+^), memory of IgM (IgM^+^IgD^-^), marginal Zone (MZ, IgM^+^IgD^+^CD27^+^), memory of IgD (IgM^-^IgD^+^CD27^+^), naïve (IgD^+^CD27^-^/CD21^+^ or CD21^-^). Class-switched cells, IgM^-^IgD^-^, were IgG^+^ or IgA^+^ or IgG^-^IgA^-^, expressing or not CD21 and CD27: activated memory (AM, CD21^-^CD27^+^), atypical memory (AtyM, CD21^-^CD27^-^), intermediate memory (IntM, CD21^+^CD27^-^) and resting memory (RM, CD21^+^CD27^+^). **(B–E)** Percentage of CD19^+^ cell subsets in healthy donors (CTL, n = 13), asymptomatic (A, n = 08), mild (B1, n = 12), and moderate/severe CCC (B2/C/D, n = 08) were defined by supervised analysis. Box and whiskers contain minimum and maximum values, median and interquartile range, and superimposed symbols represent individual values. Asterisks represent significant differences between the assigned groups. *p < 0.05, **p < 0.01, ***p < 0.001, ****p < 0.0001.

Distinct T cell subsets differently impact the development and differentiation of B cells. We assessed the composition of B cell compartments based on most of the peripheral B cell differentiation stages. B cells were defined as CD19^+^CD20^+^CD21^+^, while plasma cells were defined as CD19^+^ lacking CD21, CD20, IgM, and IgD but expressing CD27 (**Figure 6A**, **Supplementary Figure 3**). From B cells, we defined transitional B cells (CD10^+^IgD^+^) and mature B cells, which were further divided into unswitched and class-switched cells based on the expression of IgM and IgD. Class-switched B cells (IgM^-^IgD^-^) were further grouped based on IgG^+^, IgA^+^ or IgG^-^IgA^-^, and each of these isotype-expressing switched B cells consists of Activated Memory (AM, CD21^-^CD27^+^), Atypical Memory (AtyM, CD21^-^CD27^-^), Intermediary Memory (IntM, CD21^+^CD27^-^) and Resting Memory (RM, CD21^+^CD27^+^) (**Figure 6A**). Unswitched cells were classified as IgM memory (IgM^+^IgD^-^), marginal zone (MZ, IgM^+^IgD^+^CD27^+^), IgD memory (IgM^-^IgD^+^CD27^+^), and naïve (IgD^+^CD27^-^CD21^+^ or CD21^-^) (**Figure 6A**, **Supplementary Figure 3**).

Chagas disease triggers the expansion of AM B cells in asymptomatic and mild CCC patients, independently of the Ig isotype (**Figure 6B**). In addition, AM IgA cells were elevated in moderate/severe CCC. Chagas patients showed higher frequencies of AtyM IgG compared to CTL. AtyM IgA and IgG^-^IgA^-^ B cells display higher frequencies in mild CCC and asymptomatic patients, respectively, compared to CTL (**Figure 6B**). No differences occurred in the frequencies of IgM and IgD memory B cells (**Figure 6C**), naïve CD21^-^ (**Figure 6D**), marginal zone, and plasma cells (**Figure 6E**). However, naïve CD21^+^ (**Figure 6D**) and transitional (**Figure 6E**) B cells were decreased in patients with mild and moderate/severe CCC, respectively, compared to CTL.

### Patients with chagas disease show heterogeneity of B cell subsets

**Figure 7:**
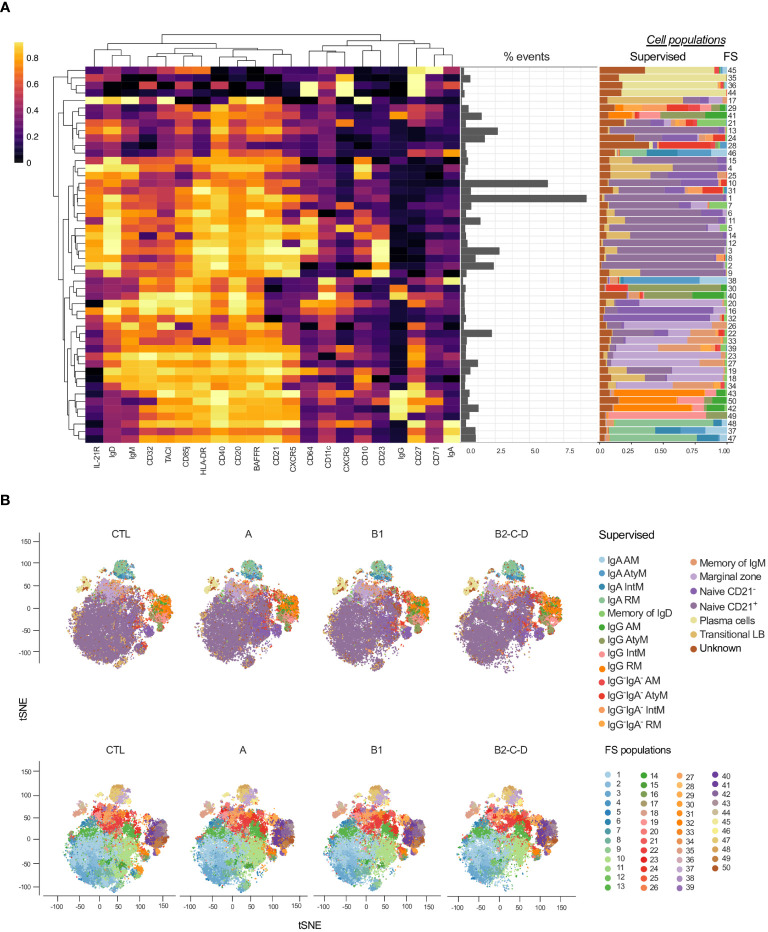
Overlay of CD19^+^ cell subsets obtained by supervised and unsupervised analyses. **(A)** Heatmap created considering the expression of 21 surface antigens (x-axis), normalized by each marker, across 50 FS populations of CD19^+^ cells distributed based on hierarchical clustering by similarity. Proportion (middle panel) and contribution of manually defined populations for each FS (right panel). **(B)** tSNE, normalized to represent the same number of events in each group, depicts manually/supervised (top panel) and unsupervised/FS (bottom panel) defined CD19^+^ cell subsets. Healthy donors (CTL, n = 13), asymptomatic (A, n = 08), mild (B1, n = 12), and moderate/severe CCC (B2/C/D, n = 08). Different colors are assigned for each supervised defined (left panel) and FS (right panel) subpopulations.

As for T cells (**Figure 2A**), we applied FlowSOM to explore distinct B cell phenotypes in patients with Chagas diseases (**Figure 7**). We thus identified several heterogeneous clusters consisting of plasma cell (FS 45-44), transitional (FS7), and class-switched (FS29-46) B cell subsets (**Figure 7A**, **Supplementary Figure 3**). Furthermore, FS15-9 predominantly represent CD21^+^ naïve B cells while FS16 and 32 are CD21^-^ naïve B cells. FS20, 26-34 are mostly constituted of marginal zone B cells. FS43-49 and FS48-47 predominantly consist of IgG^+^ and IgA^+^ B cells, respectively (**Figure 7A**).

In the dimensionality reduction obtained by supervised (**Figure 7B**, top panel) analysis, we observed an increase in the frequency of class-switched subsets: in IgG, IgA, and IgG^-^IgA^-^ AM and AtyM B cells with the progression of Chagas disease (groups A, B1 and B2/C/D). On the other hand, naïve and transitional B cells were decreased in patients with CCC compared to CTL, especially in moderate/severe CCC group. tSNE generated with the FS populations (**Figure 7B**, bottom panel) revealed several differences among the clinical forms and CTL (**Figure 7B**, bottom panel).

### Chagas disease triggers the expansion of activated and memory B cells and the reduction of their naïve subsets

**Figure 8:**
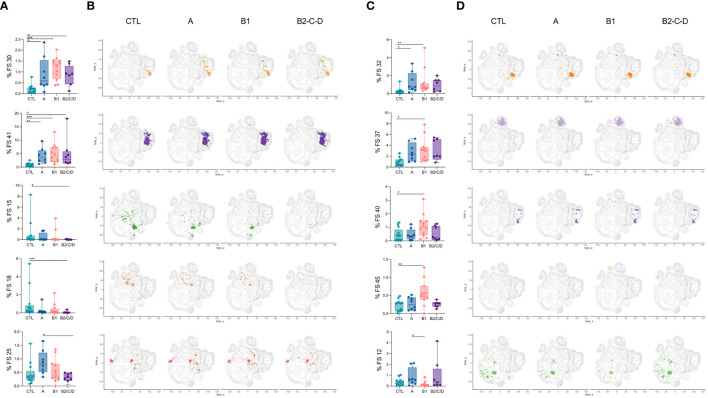
Patients with Chagas disease have expanded FS populations of CD19+ cells with activated and memory phenotypes. Percentages of FS 30, 41, 15, 18, and 25 **(A)**, and 32, 37, 40, 45, and 12 **(C)** within CD19^+^ cells (from top to bottom) in healthy donors (CTL, n = 13), asymptomatic (A, n = 08), mild (B1, n = 12), and moderate/severe CCC (B2/C/D, n = 08). Box and whiskers contain minimum and maximum values, median and interquartile range, and superimposed symbols representing individual values. Asterisks represent significant differences between the assigned groups. *p < 0.05, **p < 0.01, ***p < 0.001. **(B, D)** tSNE contour plots show CD19^+^ cells in gray and FS populations in colors, as in **Figure 7B**, from CTL and infected patients in different stages of Chagas disease.

Eight FS were altered in at least one of the clinical forms compared to CTL; one was altered between asymptomatic and mild CCC patients, and another was altered between asymptomatic and moderate/severe CCC (**Figure 8**). Forty FS were similar among groups (**Supplementary Figure 4**). Higher frequencies of FS 30 and 41 were observed in Chagas patients compared to CTL, regardless of the clinical form (**Figures 8A, B**). Both FS populations are composed of AtyM cells, but express different levels of co-receptors and activation markers such as CD20, CD85j, CD40, TACI, CD32, among others (**Supplementary Figures 5, 6**). Moderate/severe CCC patients have lower frequencies of FS15 and FS18 than CTL and FS25 than asymptomatic patients. Those are composed of naïve and transitional cells. FS15 expresses high levels of IgD and intermediate levels of CD20, CD40, and HLA-DR; FS25 expresses high levels of IgM and IL-21R and intermediate levels of CD20, CD40 and BAFFR; and FS18 expresses high levels of both IgM and IgD, CD20, CD40, CD32, HLA-DR, BAFFR and TACI, and does not express IL-21R (**Figures 8A, B**, **Supplementary Figure 6**).

FS32 was expanded in asymptomatic and mild CCC patients compared to CTL and was characterized by the expression of IgD and high levels of CD20, HLA-DR, CD85j, and CD32 (**Figures 8C, D**, **Supplementary Figure 6**).

FS37, 40, and 45 were exclusively increased in mild CCC compared to CTL. FS37 consists mostly of IgA cells and comprises AM, AtyM, RM, and IntM cells. This cluster is characterized by intermediate levels of BAFFR, CD20, HLA-DR and CD40. FS40 contains IgG-expressing AM and AtyM cells and displays high levels of CD20, CD85j, CD11c, TACI, HLA-DR, CD32, and intermediate levels of BAFFR and CD40. FS45 represents plasma cells expressing high levels of CD27 and CD71. The only FS that decreased in mild CCC compared to CTL was FS12, a naïve subset with increased expression of IgD, CD40, HLA-DR, CD23, BAFFR, and CD64 (**Figures 8C, D**, **Supplementary Figure 6**).

In general, Chagas disease leads to expansion of activated, class-switched B cell subsets, which is more prominent in mild CCC clinical form.

## Discussion

The progression of Chagas disease is determined by the balance between the host’s immune response and the dynamics of the *T. cruzi* parasite replication (6, 42). It is well established that an environment with balanced pro- and anti-inflammatory immune responses is associated with an asymptomatic chronic course of the disease. In contrast, an excessive pro-inflammatory response can result in cardiac pathology (42). An ineffective immunological response can exacerbate the parasitic burden and incite an overwhelming inflammatory response, leading to tissue damage (43). Conversely, in the case of an effective immune response, the parasitic load and the inflammatory consequences are minimized (44).

Upon infection, mammalian hosts develop adaptive immunity that plays a significant role during the chronic phases of the disease (18). The specific immune response, particularly CD8^+^ and CD4^+^ T cells against *T. cruzi*, are relevant for parasite control and disease pathogenesis (45, 46). Despite the acknowledged role of cytotoxic T cells in the pathogenesis of Chagas disease (45), the contributions of CD4^+^ T cells and B cells are less understood, likely due to their ability to interfere with other immune cells’ function (18). Therefore, a comprehensive analysis of CD4^+^ T cell and B cell subsets across distinct clinical forms of Chagas disease is crucial. Our employed multidimensional flow cytometry approaches to characterize these subsets, aiming to elucidate their role in the disease’s pathogenesis.

Our findings show that Chagas disease alters circulating CD4^+^ T cell compartments. Patients with both mild (B1) and moderate/severe (B2/C/D) CCC exhibited an increased frequency of effector CD27^-^ cells. Interestingly, a lower frequency of CM among cells expressing both CD45RA and CCR7 was observed in patients with moderate/severe CCC. In contrast, a decrease in CM cells was noted in patients with asymptomatic form (A) compared to CTL. Patients with mild CCC demonstrated an expansion of EM compared to CTL. These data corroborate the findings from Fiuza and collaborators (2009) (47), reporting that patients with the asymptomatic clinical form of the disease had more EM CD4^+^ T cells, which may induce a regulatory mechanism to protect the host against the exacerbated inflammatory response caused by the infection. However, in contrast to our data, the same authors demonstrated that patients with asymptomatic clinical form had more CM CD4^+^ T cells than healthy individuals. This discrepancy may be owing to the fact that memory profile of Chagas’ patients was evaluated after *in vitro T. cruzi*- stimulation.

Unsupervised analysis reinforced the heterogeneity of CD4^+^ T cell subsets and revealed an effector and activated profile of CD4^+^ T cells in patients with chronic Chagas disease. Previous research has identified that patients with mild chronic Chagas disease exhibit an expansion in activated of CD4^+^ T cells, and high frequencies of IFN-γ, IFN-γ^+^TNF^+^ and of IFN-γ^+^TNF^+^CD154^+^ among EM CD4^+^ T cells compared to CTL (48). These findings suggest a potential role for these cells in the establishment of cardiac lesions and their potential utility as biomarkers for monitoring disease progression. Aligning with these data, our study has revealed an expanded frequency of Eff CD27^-^ and EM CD4^+^ T cells in the mild CCC group, which differed from the asymptomatic group. Taken together, the stratification of Eff CD4^+^ T cells may represent a new marker of the clinical progression of Chagas disease.

Expansion of Tfh cells was observed in mild CCC patients compared to asymptomatic patients. A previous study demonstrated various circulating CD4^+^CD45RO^+^CXCR5^+^ cell subsets in patients with distinct clinical forms of chronic Chagas disease (30). The expansion of CCR6^+^ Tfh cells and decreased Th2-like Tfh cells was shown, regardless of their clinical status. Other phenotypic changes included an increase of Th17 and a decrease of Th1-like Tfh cells in asymptomatic patients but not in those with CCC. On the other hand, other studies show decrease in levels of IL-17 and proportions of IL-17 producing CD4^+^ T cells in CCC compared to asymptomatic patients and CTL (31, 49). Although these findings are controversial, distinct Tfh phenotypes might contribute differently to Chagas disease progression (21, 22).

A higher proportion of Th1, senescent and/or exhausted T cells, and a lower frequency of circulating multifunctional CD4^+^ T subset in CCC patients have been associated with the progression of heart disease (50). Pérez-Anton et al., 2021 (51) found higher frequencies of CD4^+^ T cells expressing inhibitory receptors (2B4, CD160, CTLA-4, PD-1, and TIM-3) in patients with Chagas disease than in healthy donors. Furthermore, patients with cardiac manifestation exhibited expanded CD4^+^ T cells coexpressing inhibitory receptors compared to asymptomatic patients. Our analysis also revealed markers of senescence and inhibitory receptors associated with cardiac stages of the disease. High frequency of FS subsets composed of heterogeneous memory CD4^+^ T cell populations expressing high levels of PD-1, ICOS, CD28, CD95, CD57, CD122, HLA-DR, CCR5, and CXCR3 are found mostly in mild CCC patients. Moreover, the expansion and contraction of T cells expressing CCR5 and CXCR3, respectively, are associated with a worse prognosis, and levels of CXCL9 and CXCL10 are augmented during CCC and are positively correlated with disease severity (52). Mild CCC patients also display expansion of another heterogeneous FS subset composed of Tfh, transitional, and CM cells that express high levels of CXCR5, PD-1, ICOS, and CD28, compared to asymptomatic patients. These findings support the hypothesis that chronic *T. cruzi* infection maintains a pool of highly activated CD4^+^ T cells, which is more exacerbated in those patients with cardiac symptoms and may lead to the attrition of a long-term memory response (9, 50, 53).

It was recently reported that CCC patients display expansion of total and transitional B cells expressing high levels of CD24 and CD38 (54). In our hand, transitional B cells were decreased in moderate/severe form of CCC. This discrepancy was probably due to different approaches applied to analyze B cells. However, corroborating with Girard et al, 2021 (54), we detect alterations in the peripheral B cell compartment from *T. cruzi*-infected patients, mainly in AM and AtyM B cell subsets.Expansion of class-switched IgA^+^ AM and IgG^+^ AtyM B cells expressing high levels of CD20, CD85j, CD11c, TACI, HLA-DR, CD32, and intermediate levels of BAFFR and CD40 were observed in all clinical forms, and more substantially in mild CCC patients. In fact, AtyM B cells seem to be associate with chronic diseases and are less functional than other memory subsets (35).

IgG antibody is considered crucial for the evolution of Chagas disease, controlling infection through the formation of microaggregates of parasites, complement opsonization, and platelet activation, which facilitate parasite internalization by phagocytic cells (55). Indeed, IgG Fc receptors (Fc-γR) are essential during the acute and chronic phase of Chagas disease, being implicated in degranulation, cytokine production, and antibody-dependent cellular cytotoxicity, and trigger activation and inhibition of pathways necessary for generating an effective immune response (56, 57). A former study employing a murine acute *T. cruzi* infection revealed an upregulation and release of CD32 (Fc-γRII) (58). During chronic Chagas disease, patients with the cardiac form have lower expression of CD32 on B cells, when compared to non-infected individuals (57). However, this study did not further classify CCC in distinct clinical stages. Our data show that patients with moderate/severe CCC have lower and mild CCC have higher frequencies of CD32 expressing B cells than CTL. Thus, these data suggest that this Fc- γR is involved in the progression of the CCC.

Altogether, our multidimensional analysis reveals highly activated T and B cell signatures associated with Chagas disease, which are more prominent in mild CCC.

This study has a few limitations, including the small number of patients with moderate/severe CCC clinical form, which may have restrained the analysis concerning how the phenotypic features related to the evaluated cells vary with the progression of the disease. Further research is required to uncover the functional effects exerted by these cell subsets during complex responses against *T. cruzi*.

## Conclusion

**Figure 9:**
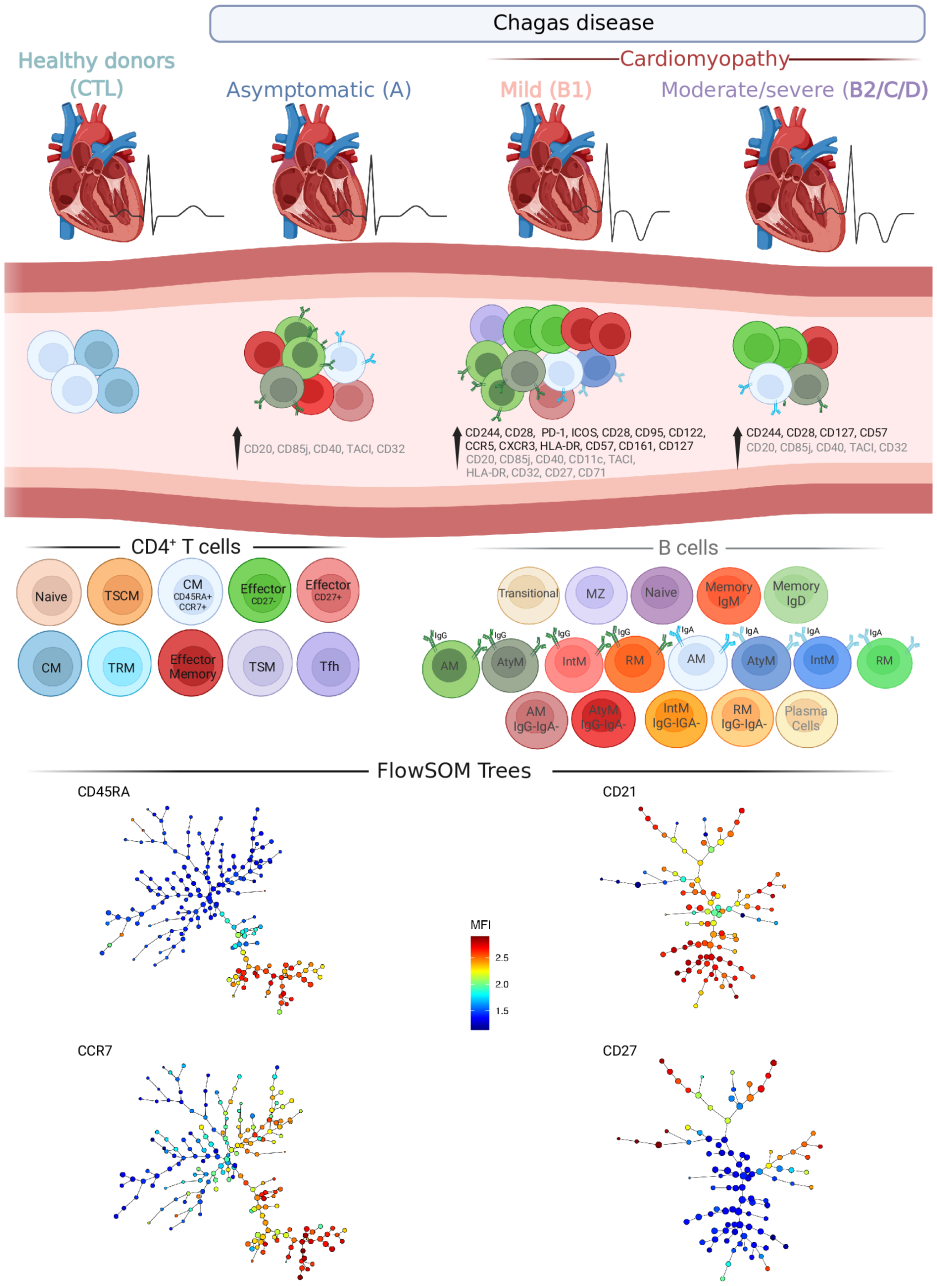
Overview of T and B cell compartments in patients with Chagas disease. Schematic drawing summarizing the most important changes in T and B cell subsets from patients in different stages of Chagas disease. Significant changes in ECG and left ventricle diameter (top) and in CD4^+^ T and B cell subsets and molecules (middle) induced by Chagas disease are represented in columns in each clinical group: Asymptomatic (A), Mild cardiomyopathy (B1), Moderate/severe cardiomyopathy (B2/C/D). Healthy donors (CTL) are shown as reference. CD4^+^ T cells and their subsets are represented with different colors and sources in black, from left to right): Naïve, stem cell-like memory (TSCM), Central Memory (CM among CD45RA^+^CCR7^+^), Effector (Eff CD27^-^ and CD27^+^), CM, tissue-resident memory (TRM), Effector memory (EM), transitional memory (TSM) and Follicular helper T (Tfh). B cells are represented with different colors and sources in gray, from left to right. Unswitched B cells: Transitional, Marginal zone, Naïve, Memory (expressing IgM or IgD). Switched B cells: IgG^+^ activated memory (AM) and IgG^+^ atypical memory (AtyM); IgA^+^AM and IgA^+^AtyM; and IgG^−^IgA^-^AM and IgG^−^IgA^-^AtyM. Plasma cells. Minimum spanning trees of CD4^+^ T (left bottom) and B cells (right bottom) represent the distribution of 50 FS populations and show the differential expression, from lower (blue) to higher (red), of respectively CD45RA and CCR7, and CD21 and CD27. Created with BioRender.com.

In summary, our study indicates that alterations of effector and memory cell phenotypes are found in patients with Chagas disease and may contribute to an inflammatory environment, characterized by highly differentiated CD4^+^ T cells with an exhaustion profile and class-switched B cells in symptomatic patients (**Figure 9**). These findings reinforce the complexity of the immune response in patients with chronic Chagas disease and provide new insights into disease pathology, shedding light on potential markers to guide clinical decisions.

## Data availability statement

The original contributions presented in the study are included in the article/**Supplementary Material**. Further inquiries can be directed to the corresponding authors.

## Ethics statement

The studies involving humans were approved by Ethical Committee on Human Research at René Rachou Institute, Oswaldo Cruz Foundation. The studies were conducted in accordance with the local legislation and institutional requirements. The participants provided their written informed consent to participate in this study.

## Author contributions

IV: Writing – review & editing, Writing – original draft, Methodology, Formal analysis. GA: Writing – review & editing, Visualization, Validation, Formal analysis. IR: Writing – review & editing, Validation, Methodology, Investigation, Formal analysis, Conceptualization. TL: Writing – review & editing, Investigation, Conceptualization. FD: Writing – review & editing, Writing – original draft, Methodology. LS: Writing – review & editing, Writing – original draft, Methodology. PH: Writing – review & editing, Methodology. ManR: Writing – review & editing, Supervision, Investigation. SE-S: Writing – review & editing, Supervision, Investigation. OM: Writing – review & editing, Resources, Funding acquisition. MarR: Writing – review & editing, Supervision, Resources, Funding acquisition, Conceptualization. AS: Writing – review & editing, Supervision, Resources, Funding acquisition. DJ: Writing – review & editing, Resources, Funding acquisition. AT-C: Writing – review & editing, Writing – original draft, Supervision, Resources, Investigation, Funding acquisition, Data curation, Conceptualization. LA: Writing – review & editing, Writing – original draft, Visualization, Validation, Resources, Project administration, Methodology, Investigation, Funding acquisition, Formal analysis, Data curation, Conceptualization.

## References

[1] Chagas disease. Organização Pan-Americana da Saúde (2023). Available at: https://www.paho.org/en/topics/chagas-disease.

[2] Chagas disease. World Health Organization (2023). Available at: https://www.who.int/health-topics/chagas-disease#tab=tab_1.

[3] Dias JC Ramos AN Jr Gontijo ED Luquetti A Shikanai-Yasuda MA Coura JR. 2 nd Brazilian consensus on chagas disease, 2015. Rev Soc Bras Med Trop. (2016) 49Suppl 1:3–60. doi: 10.1590/0037-8682-0505-2016 27982292

[4] Macaluso G Grippi F Di Bella S Blanda V Gucciardi F Torina A. A Review on the Immunological Response against *Trypanosoma cruzi* . Pathogens. (2023) 12:1–15. doi: 10.3390/pathogens12020282 PMC9964664 36839554

[5] Tanowitz HB MaChado FS Spray DC Friedman JM Weiss OS Lora JN. Developments in the management of Chagas cardiomyopathy. Expert Rev Cardiovasc Ther. (2015) 13:1393–409. doi: 10.1586/14779072.2015.1103648 PMC4810774 26496376

[6] Torres RM Correia D Nunes M Dutra WO Talvani A Sousa AS. Prognosis of chronic Chagas heart disease and other pending clinical challenges. Mem Inst Oswaldo Cruz. (2022) 117:e210172. doi: 10.1590/0074-02760210172 35674528 PMC9172891

[7] Martins-Melo FR Ramos Junior AN Alencar CH Heukelbach J. Multiple causes of death related to Chagas' disease in Brazil, 1999 to 2007. Rev Soc Bras Med Trop. (2012) 45:591–6. doi: 10.1590/S0037-86822012000500010 23152342

[8] Puerta CJ Cuellar A Lasso P Mateus J Gonzalez JM. *Trypanosoma cruzi*-specific CD8(+) T cells and other immunological hallmarks in chronic Chagas cardiomyopathy: Two decades of research. Front Cell Infect Microbiol. (2022) 12:1075717. doi: 10.3389/fcimb.2022.1075717 36683674 PMC9846209

[9] Arguello RJ Vigliano C Cabeza-Meckert P Viotti R Garelli F Favaloro LE. Presence of antigen-experienced T cells with low grade of differentiation and proliferative potential in chronic Chagas disease myocarditis. PloS Negl Trop Dis. (2014) 8:e2989. doi: 10.1371/journal.pntd.0002989 25144227 PMC4140664

[10] Higuchi Mde L Gutierrez PS Aiello VD Palomino S Bocchi E Kalil J. Immunohistochemical characterization of infiltrating cells in human chronic chagasic myocarditis: comparison with myocardial rejection process. Virchows Arch A Pathol Anat Histopathol. (1993) 423:157–60. doi: 10.1007/BF01614765 7901937

[11] Reis DD Jones EM Tostes S Jr. Lopes ER Gazzinelli G Colley DG. Characterization of inflammatory infiltrates in chronic chagasic myocardial lesions: presence of tumor necrosis factor-alpha+ cells and dominance of granzyme A+, CD8+ lymphocytes. Am J Trop Med Hyg. (1993) 48:637–44. doi: 10.4269/ajtmh.1993.48.637 8517482

[12] Bahia-Oliveira LM Gomes JA Rocha MO Moreira MC Lemos EM Luz ZM. IFN-gamma in human Chagas' disease: protection or pathology? Braz J Med Biol Res. (1998) 31:127–31. doi: 10.1590/S0100-879X1998000100017 9686189

[13] Dutra WO Menezes CA Villani FN da Costa GC da Silveira AB Reis D. Cellular and genetic mechanisms involved in the generation of protective and pathogenic immune responses in human Chagas disease. Mem Inst Oswaldo Cruz. (2009) 104 Suppl 1:208–18. doi: 10.1590/S0074-02762009000900027 PMC3285444 19753476

[14] Gomes JA Bahia-Oliveira LM Rocha MO Martins-Filho OA Gazzinelli G Correa-Oliveira R. Evidence that development of severe cardiomyopathy in human Chagas' disease is due to a Th1-specific immune response. Infect Immun. (2003) 71:1185–93. doi: 10.1128/IAI.71.3.1185-1193.2003 PMC148818 12595431

[15] de Araujo FF Vitelli-Avelar DM Teixeira-Carvalho A Antas PR Assis Silva Gomes J Sathler-Avelar R. Regulatory T cells phenotype in different clinical forms of Chagas' disease. PloS Negl Trop Dis. (2011) 5:e992. doi: 10.1371/journal.pntd.0000992 21655351 PMC3104959

[16] Vitelli-Avelar DM Sathler-Avelar R Mattoso-Barbosa AM Gouin N Perdigao-de-Oliveira M Valerio-Dos-Reis L. Cynomolgus macaques naturally infected with *Trypanosoma cruzi*-I exhibit an overall mixed pro-inflammatory/modulated cytokine signature characteristic of human Chagas disease. PloS Negl Trop Dis. (2017) 11:e0005233. doi: 10.1371/journal.pntd.0005233 28225764 PMC5321273

[17] Acevedo GR Girard MC Gomez KA. The unsolved jigsaw puzzle of the immune response in chagas disease. Front Immunol. (2018) 9:1929. doi: 10.3389/fimmu.2018.01929 30197647 PMC6117404

[18] Cardillo F Postol E Nihei J Aroeira LS Nomizo A Mengel J. B cells modulate T cells so as to favour T helper type 1 and CD8+ T-cell responses in the acute phase of *Trypanosoma cruzi* infection. Immunology. (2007) 122:584–95. doi: 10.1111/j.1365-2567.2007.02677.x PMC2266037 17635611

[19] Sullivan NL Eickhoff CS Sagartz J Hoft DF. Deficiency of antigen-specific B cells results in decreased *Trypanosoma cruzi* systemic but not mucosal immunity due to CD8 T cell exhaustion. J Immunol. (2015) 194:1806–18. doi: 10.4049/jimmunol.1303163 PMC4324165 25595788

[20] Fernandez ER Olivera GC Quebrada Palacio LP Gonzalez MN Hernandez-Vasquez Y Sirena NM. Altered distribution of peripheral blood memory B cells in humans chronically infected with Trypanosoma cruzi. PloS One. (2014) 9:e104951. doi: 10.1371/journal.pone.0104951 25111833 PMC4128741

[21] Minoprio P Burlen O Pereira P Guilbert B Andrade L Hontebeyrie-Joskowicz M. Most B cells in acute *Trypanosoma cruzi* infection lack parasite specificity. Scand J Immunol. (1988) 28:553–61. doi: 10.1111/j.1365-3083.1988.tb01487.x 2463663

[22] Bryan MA Guyach SE Norris KA. Specific humoral immunity versus polyclonal B cell activation in *Trypanosoma cruzi* infection of susceptible and resistant mice. PloS Negl Trop Dis. (2010) 4:e733. doi: 10.1371/journal.pntd.0000733 20625554 PMC2897841

[23] Bermejo DA Amezcua Vesely MC Khan M Acosta Rodriguez EV Montes CL Merino MC. *Trypanosoma cruzi* infection induces a massive extrafollicular and follicular splenic B-cell response which is a high source of non-parasite-specific antibodies. Immunology. (2011) 132:123–33. doi: 10.1111/j.1365-2567.2010.03347.x PMC3015082 20875075

[24] Cunha-Neto E Teixeira PC Nogueira LG Kalil J. Autoimmunity. Adv Parasitol. (2011) 76:129–52. doi: 10.1016/B978-0-12-385895-5.00006-2 21884890

[25] Breitfeld D Ohl L Kremmer E Ellwart J Sallusto F Lipp M. Follicular B helper T cells express CXC chemokine receptor 5, localize to B cell follicles, and support immunoglobulin production. J Exp Med. (2000) 192:1545–52. doi: 10.1084/jem.192.11.1545 PMC2193094 11104797

[26] Schaerli P Willimann K Lang AB Lipp M Loetscher P Moser B. CXC chemokine receptor 5 expression defines follicular homing T cells with B cell helper function. J Exp Med. (2000) 192:1553–62. doi: 10.1084/jem.192.11.1553 PMC2193097 11104798

[27] Nurieva RI Chung Y Hwang D Yang XO Kang HS Ma L. Generation of T follicular helper cells is mediated by interleukin-21 but independent of T helper 1, 2, or 17 cell lineages. Immunity. (2008) 29:138–49. doi: 10.1016/j.immuni.2008.05.009 PMC2556461 18599325

[28] Boswell KL Paris R Boritz E Ambrozak D Yamamoto T Darko S. Loss of circulating CD4 T cells with B cell helper function during chronic HIV infection. PloS Pathog. (2014) 10:e1003853. doi: 10.1371/journal.ppat.1003853 24497824 PMC3911819

[29] Ueno H Banchereau J Vinuesa CG. Pathophysiology of T follicular helper cells in humans and mice. Nat Immunol. (2015) 16:142–52. doi: 10.1038/ni.3054 PMC4459756 25594465

[30] Quebrada Palacio LP Fernandez ER Hernandez-Vasquez Y Petray PB Postan M. Circulating T follicular helper cell abnormalities associated to different clinical forms of chronic Chagas disease. Front Cell Infect Microbiol. (2020) 10:126. doi: 10.3389/fcimb.2020.00126 32296649 PMC7136390

[31] Souza-Silva TG Neves EGA Koh C Teixeira-Carvalho A Araujo SS Nunes M. Correlation of blood-based immune molecules with cardiac gene expression profiles reveals insights into Chagas cardiomyopathy pathogenesis. Front Immunol. (2024) 15:1338582. doi: 10.3389/fimmu.2024.1338582 38390336 PMC10882095

[32] Beddall M Chattopadhyay PK Kao SF Foulds K Roederer M. A simple tube adapter to expedite and automate thawing of viably frozen cells. J Immunol Methods. (2016) 439:74–8. doi: 10.1016/j.jim.2016.08.009 27594593

[33] Liechti T Van Gassen S Beddall M Ballard R Iftikhar Y Du R. A robust pipeline for high-content, high-throughput immunophenotyping reveals age- and genetics-dependent changes in blood leukocytes. Cell Rep Methods. (2023) 3:100619. doi: 10.1016/j.crmeth.2023.100619 37883924 PMC10626267

[34] Liechti T Roederer M. OMIP-058: 30-parameter flow cytometry panel to characterize iNKT, NK, unconventional and conventional T cells. Cytometry A. (2019) 95:946–51. doi: 10.1002/cyto.a.23850 31334918

[35] Liechti T Roederer M. OMIP-051 - 28-color flow cytometry panel to characterize B cells and myeloid cells. Cytometry A. (2019) 95:150–5. doi: 10.1002/cyto.a.23689 PMC6546165 30549419

[36] Van Gassen S Callebaut B Van Helden MJ Lambrecht BN Demeester P Dhaene T. FlowSOM: Using self-organizing maps for visualization and interpretation of cytometry data. Cytometry A. (2015) 87:636–45. doi: 10.1002/cyto.a.22625 25573116

[37] Straub C Neulen ML Viertlboeck BC Gobel TW. Chicken SLAMF4 (CD244, 2B4), a receptor expressed on thrombocytes, monocytes, NK cells, and subsets of alphabeta-, gammadelta- T cells and B cells binds to SLAMF2. Dev Comp Immunol. (2014) 42:159–68. doi: 10.1016/j.dci.2013.09.007 24055739

[38] Agresta L Hoebe KHN Janssen EM. The emerging role of CD244 signaling in immune cells of the tumor microenvironment. Front Immunol. (2018) 9:2809. doi: 10.3389/fimmu.2018.02809 30546369 PMC6279924

[39] d'Angeac AD Monier S Pilling D Travaglio-Encinoza A Reme T Salmon M. CD57+ T lymphocytes are derived from CD57- precursors by differentiation occurring in late immune responses. Eur J Immunol. (1994) 24:1503–11. doi: 10.1002/eji.1830240707 7517872

[40] Di Mitri D Azevedo RI Henson SM Libri V Riddell NE Macaulay R. Reversible senescence in human CD4+CD45RA+CD27- memory T cells. J Immunol. (2011) 187:2093–100. doi: 10.4049/jimmunol.1100978 21788446

[41] Fergusson JR Smith KE Fleming VM Rajoriya N Newell EW Simmons R. CD161 defines a transcriptional and functional phenotype across distinct human T cell lineages. Cell Rep. (2014) 9:1075–88. doi: 10.1016/j.celrep.2014.09.045 PMC4250839 25437561

[42] Magalhaes LMD Gollob KJ Zingales B Dutra WO. Pathogen diversity, immunity, and the fate of infections: lessons learned from *Trypanosoma cruzi* human-host interactions. Lancet Microbe. (2022) 3:e711–22. doi: 10.1016/S2666-5247(21)00265-2 36058233

[43] Olivo Freites C Sy H Gharamti A Higuita NIA Franco-Paredes C Suarez JA. Chronic chagas disease-the potential role of reinfections in cardiomyopathy pathogenesis. Curr Heart Fail Rep. (2022) 19:279–89. doi: 10.1007/s11897-022-00568-9 35951245

[44] Sathler-Avelar R Vitelli-Avelar DM Mattoso-Barbosa AM Pascoal-Xavier MA Eloi-Santos SM da Costa-Rocha IA. Phenotypic and functional signatures of peripheral blood and spleen compartments of cynomolgus macaques infected with T. cruzi: associations with cardiac histopathological characteristics. Front Cell Infect Microbiol. (2021) 11:701930. doi: 10.3389/fcimb.2021.701930 34336723 PMC8317693

[45] Acosta Rodriguez EV Araujo Furlan CL Fiocca Vernengo F Montes CL Gruppi A. Understanding CD8(+) T cell immunity to *trypanosoma cruzi* and how to improve it. Trends Parasitol. (2019) 35:899–917. doi: 10.1016/j.pt.2019.08.006 31607632 PMC6815727

[46] Ferragut F Acevedo GR Gomez KA. T cell specificity: A great challenge in chagas disease. Front Immunol. (2021) 12:674078. doi: 10.3389/fimmu.2021.674078 34267750 PMC8276045

[47] Fiuza JA Fujiwara RT Gomes JA Rocha MO Chaves AT de Araujo FF. Profile of central and effector memory T cells in the progression of chronic human chagas disease. PloS Negl Trop Dis. (2009) 3:e512. doi: 10.1371/journal.pntd.0000512 19742301 PMC2729721

[48] Almeida GG Rimkute I do Vale I Liechti T Henriques PM Roffe E. Chagasic cardiomyopathy is marked by a unique signature of activated CD4(+) T cells. J Transl Med. (2022) 20:551. doi: 10.1186/s12967-022-03761-5 36447264 PMC9708147

[49] de Araujo FF Correa-Oliveira R Rocha MO Chaves AT Fiuza JA Fares RC. Foxp3+CD25(high) CD4+ regulatory T cells from indeterminate patients with Chagas disease can suppress the effector cells and cytokines and reveal altered correlations with disease severity. Immunobiology. (2012) 217:768–77. doi: 10.1016/j.imbio.2012.04.008 22672991

[50] Albareda MC Olivera GC Laucella SA Alvarez MG Fernandez ER Lococo B. Chronic human infection with *Trypanosoma cruzi* drives CD4+ T cells to immune senescence. J Immunol. (2009) 183:4103–8. doi: 10.4049/jimmunol.0900852 PMC3074976 19692645

[51] Perez-Anton E Egui A Thomas MC Carrilero B Simon M Lopez-Ruz MA. A proportion of CD4+ T cells from patients with chronic Chagas disease undergo a dysfunctional process, which is partially reversed by benznidazole treatment. PloS Negl Trop Dis. (2021) 15:e0009059. doi: 10.1371/journal.pntd.0009059 33539379 PMC7888659

[52] Roffe E Dos Santos LI Santos MO Henriques PM Teixeira-Carvalho A Martins-Filho OA. Increased frequencies of circulating CCR5(+) memory T cells are correlated to chronic chagasic cardiomyopathy progression. J Leukoc Biol. (2019) 106:641–52. doi: 10.1002/JLB.MA1118-472R 31087713

[53] Albareda MC De Rissio AM Tomas G Serjan A Alvarez MG Viotti R. Polyfunctional T cell responses in children in early stages of chronic *Trypanosoma cruzi* infection contrast with monofunctional responses of long-term infected adults. PloS Negl Trop Dis. (2013) 7:e2575. doi: 10.1371/journal.pntd.0002575 24349591 PMC3861186

[54] Girard MC Acevedo GR Ossowski MS Fernandez M Hernandez Y Chadi R. Ex vivo characterization of Breg cells in patients with chronic Chagas disease. Sci Rep. (2021) 11:5511. doi: 10.1038/s41598-021-84765-x 33750870 PMC7943772

[55] Georg I Hasslocher-Moreno AM Xavier SS Holanda MT Roma EH Bonecini-Almeida MDG. Evolution of anti-*Trypanosoma cruzi* antibody production in patients with chronic Chagas disease: Correlation between antibody titers and development of cardiac disease severity. PloS Negl Trop Dis. (2017) 11:e0005796. doi: 10.1371/journal.pntd.0005796 28723905 PMC5536389

[56] Ravetch JV Lanier LL. Immune inhibitory receptors. Science. (2000) 290:84–9. doi: 10.1126/science.290.5489.84 11021804

[57] Gomes JAS de Araujo FF Vitelli-Avelar DM Sathler-Avelar R Lage PS Wendling APB. Systems biology reveals relevant gaps in Fc-gammaR expression, impaired regulatory cytokine microenvironment interfaced with anti-*trypanosoma cruzi* IgG reactivity in cardiac Chagas disease patients. Front Microbiol. (2018) 9:1608. doi: 10.3389/fmicb.2018.01608 30105007 PMC6077235

[58] Caraujo-Jorge T el Bouhdidi A Rivera MT Daeron M Carlier Y Jorge TA. *Trypanosoma cruzi* infection in mice enhances the membrane expression of low-affinity Fc receptors for IgG and the release of their soluble forms. Parasite Immunol. (1993) 15:539–46. doi: 10.1111/j.1365-3024.1993.tb00642.x 7877851

