# Supplementary material for: Signatures of CD4+ T and B cells are associated with distinct stages of chronic chagasic cardiomyopathy
Source: Front Immunol. 2024 Apr 25;15:1385850. doi: 10.3389/fimmu.2024.1385850 (PMC11079136; doi:10.3389/fimmu.2024.1385850)
Supplement: Supplementary file 1 [file DataSheet_1.pdf]

Supplementary Material – Vale et al.

Supplementay Figure 1

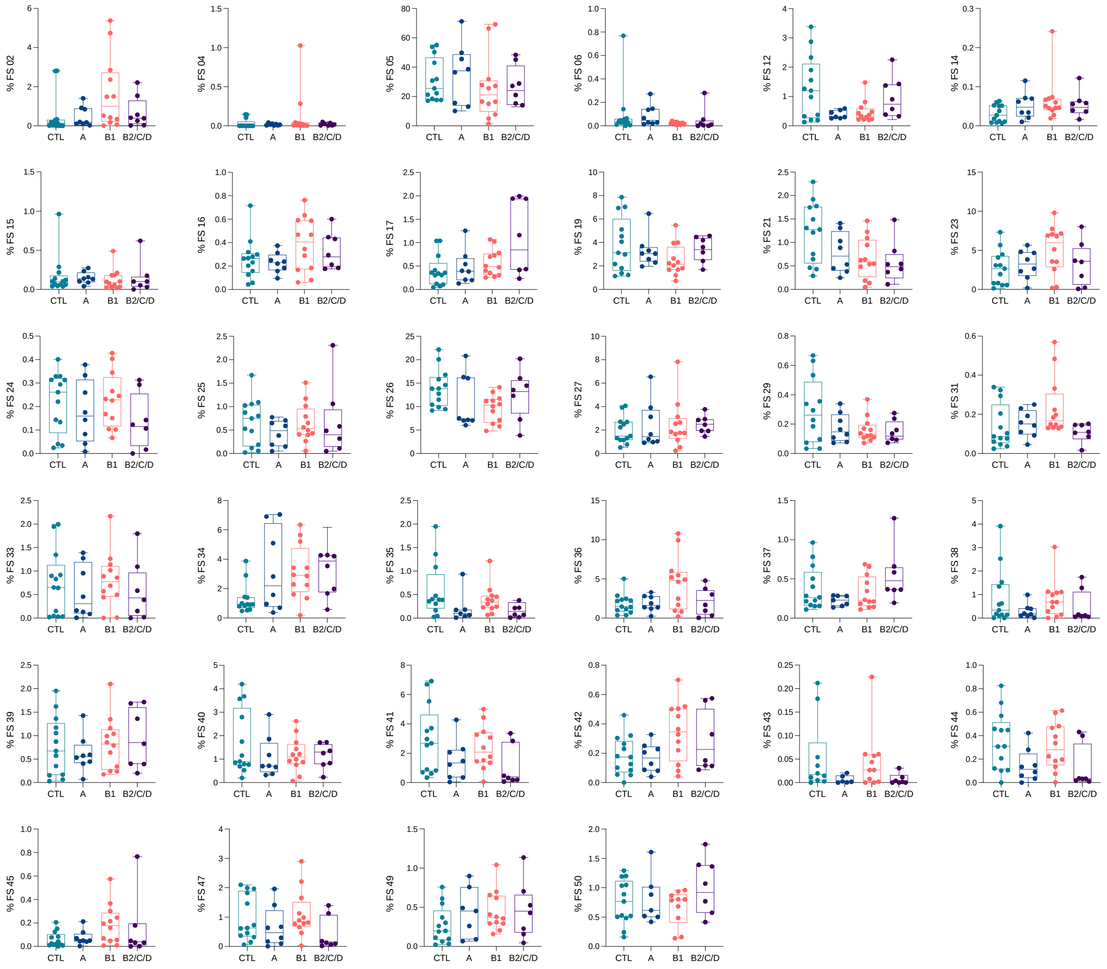

Supplementary Figure 1. FS populations of CD4⁺ T cells. Percentage of FS 02, 04-06, 12, 14-17, 19, 21, 23-27, 29, 31, 33-45, 47, 49 and 50 within CD4⁺ T cells in healthy donors (CTL, n = 13), asymptomatic (A, n = 08), mild CCC (B1, n = 12), and moderate/severe CCC (B2/C/D, n = 08). Box and whiskers containing minimum and maximum values, median and interquartile range, and superimposed symbols representing individual values. Asterisks represent significant differences between the assigned groups.

FlowSOM numbers

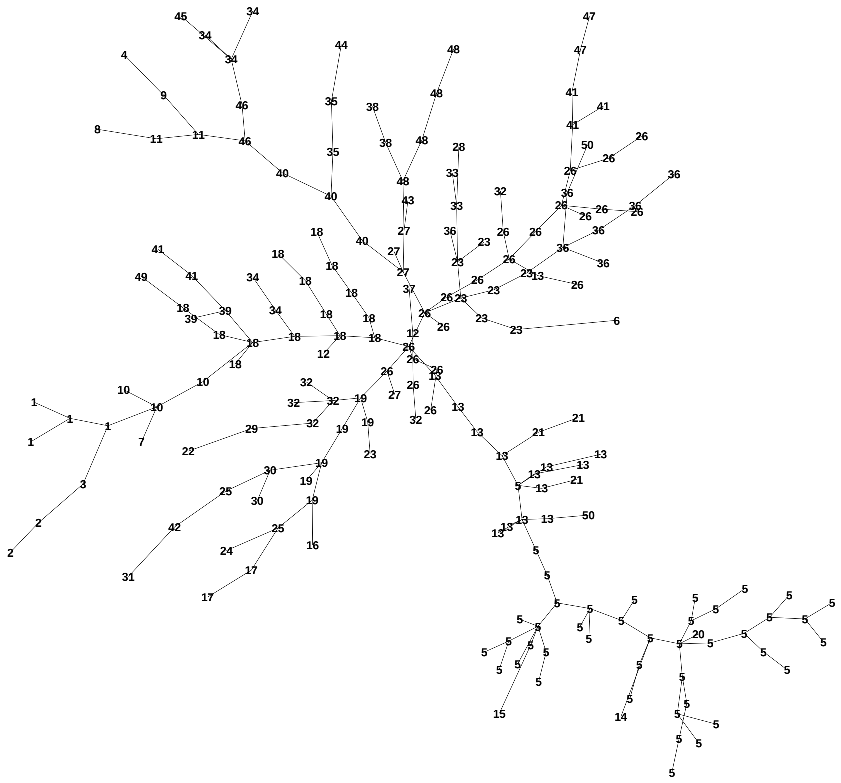

Supplementary Figure 2. Location by numbers of FS populations of CD4⁺ T cells. Minimum spanning tree (MST) composed by 196 nodes representing the distribution of FS populations of CD4⁺ T cells.

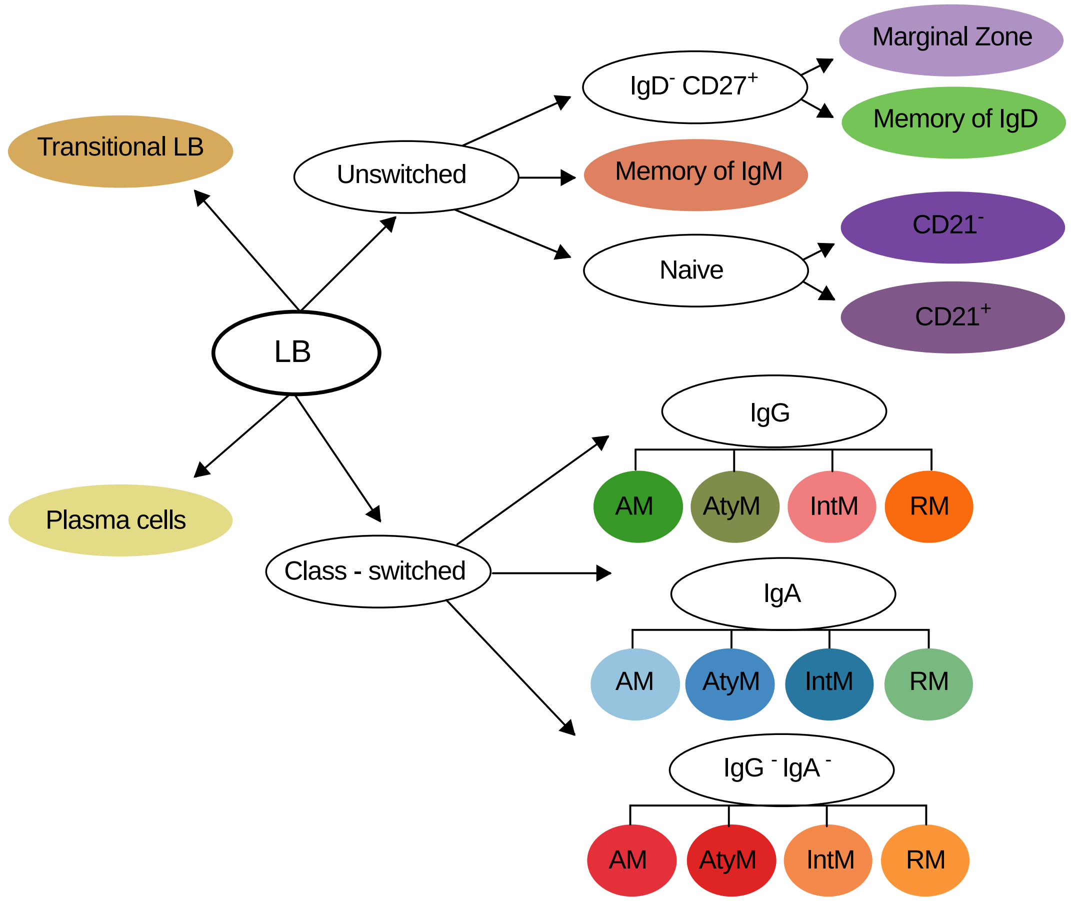

Supplementary Figure 3. Organizational chart representing B cell subsets defined by supervised analysis. Plasma cells were defined as CD21$^-$CD20$^-$IgM$^-$IgD$^-$CD27$^+$ cells. B cells (CD21$^+$CD20$^+$) and their subsets were classified according to the expression of CD10, IgD, IgM, IgG, IgA, CD27 and CD21: transitional (IgD$^+$CD10$^+$), memory of IgM (IgM$^+$IgD$^-$), marginal Zone (MZ, IgM$^+$IgD$^+$CD27$^+$), memory of IgD (IgM$^-$IgD$^+$CD27$^+$), naïve (IgD$^+$CD27$^-$/ CD21$^+$ or CD21$^-$). Class-switched cells, IgM$^-$IgD$^-$, were IgG$^+$ or IgA$^+$ or IgG$^-$IgA$^-$, expressing or not CD21 and CD27: activated memory (AM, CD21$^-$CD27$^+$), atypical memory (AtyM, CD21$^-$CD27$^-$), intermediate memory (IntM, CD21$^+$CD27$^-$) and resting memory (RM, CD21$^+$CD27$^+$).

Supplementary Figure 4

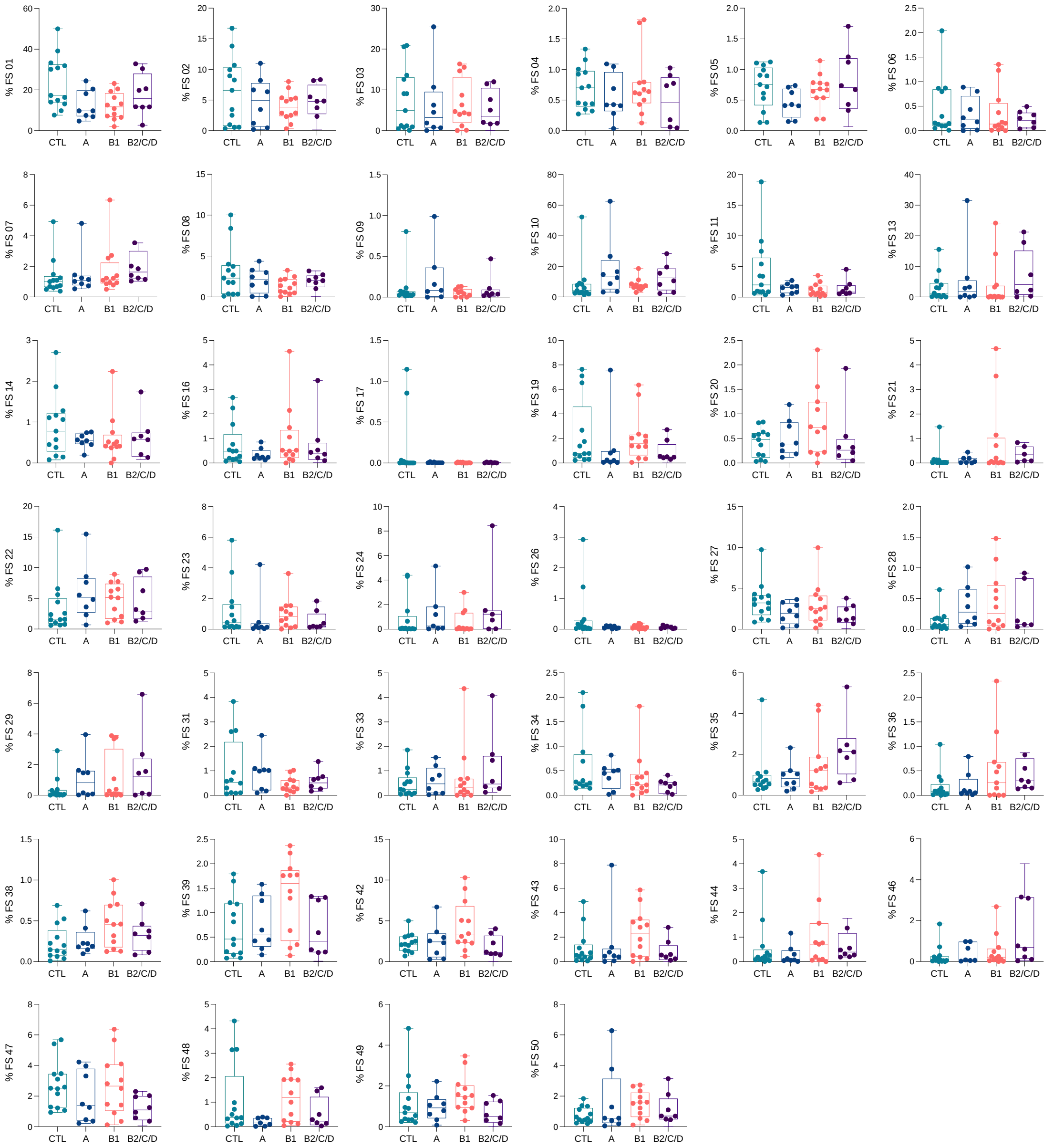

Supplementary Figure 4. FS populations of CD19⁺ cells. Percentage of FS 01-11, 13, 14, 16, 17, 19-24, 26-29, 31, 33-36, 38, 39, 42-44, 46-50 within CD19⁺ cells in healthy donors (CTL, n = 13), asymptomatic (A, n = 08), mild CCC (B1, n = 12), and moderate/severe CCC (B2/C/D, n = 08). Box and whiskers containing minimum and maximum values, median and interquartile range, and superimposed symbols representing individual values. Asterisks represent significant differences between the assigned groups.

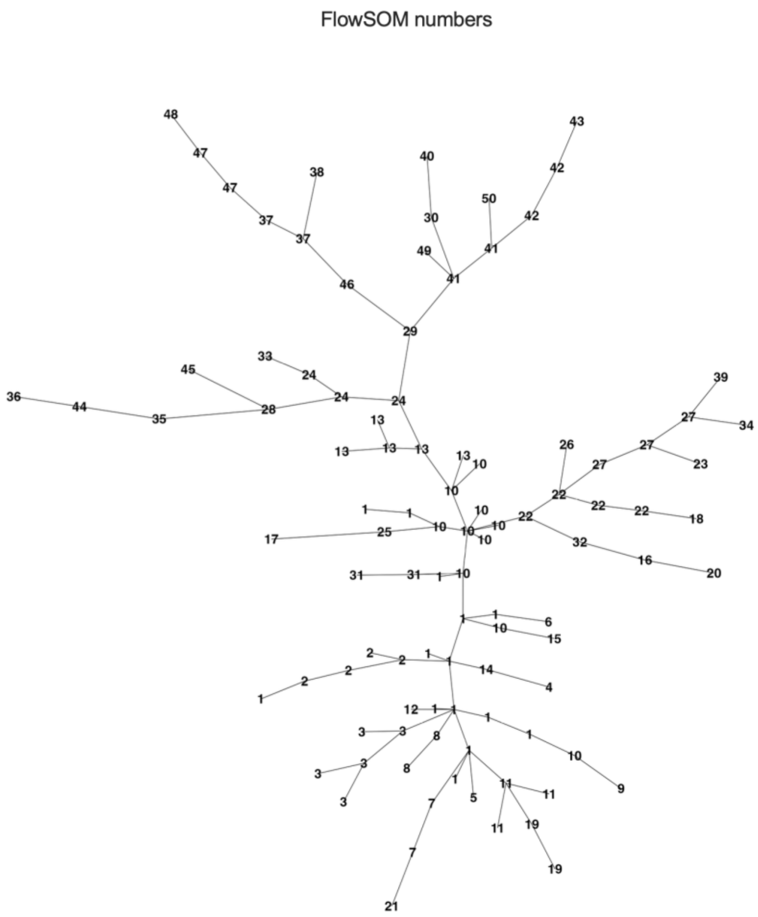

Supplementary Figure 5. Location by numbers of FS populations of CD19$^+$ cells. Minimum spanning tree (MST) composed by 196 nodes representing the distribution of FS populations of CD19$^+$ cells.

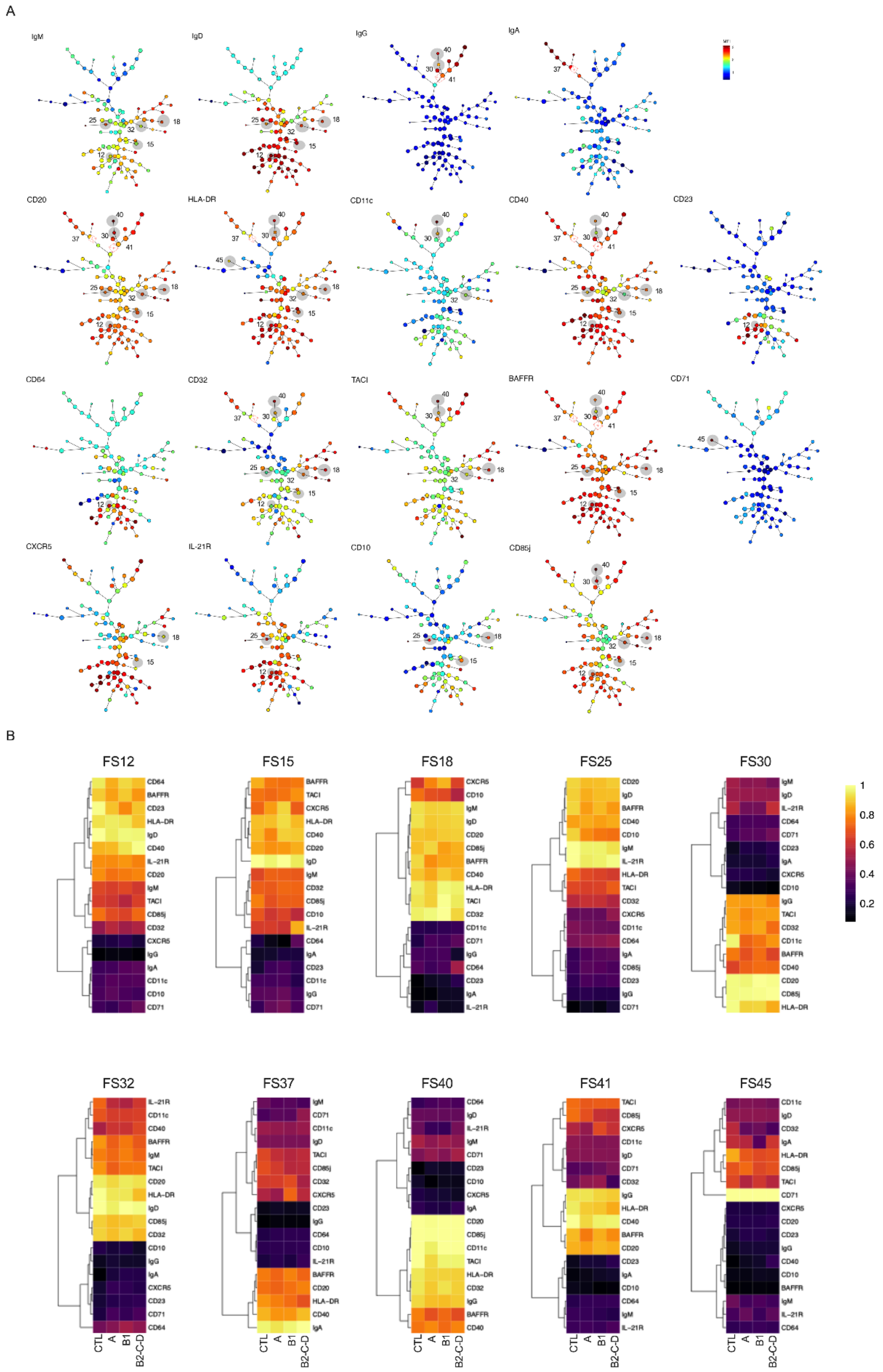

Supplementary Figure 6. Differential expression of molecules in FS populations of CD19+ cells. (A) Minimum spanning trees (MST), composed by

100 clusters representing the distribution of 50 FS populations, show the differential expression, from lower (blue) to higher (red), of IgM, IgD, IgG, IgA, CD20, HLA-DR, CD11c, CD40, CD23, CD64, CD32, TACI, BAFFR, CD71, CXCR5, IL-21R, CD10 and CD85j. FS populations, which are different between at least two groups (as in Figure 8), are delimited in dotted circles and highlighted in gray when high expression of markers is observed. (A) and (B) heatmap created considering the normalized expression of 18 surface antigens distributed based on hierarchical clustering by similarity among CTL and infected patients in different stages of Chagas disease.

Supplementary Table 1

Anamnesis of patients with Chagas disease

Clinical forms	Sex Male (%)	Age Mean ± SD	LVEF %* Mean ± SD	LV (mm)** Mean ± SD
Healthy donors (CTL)	61	45 ± 20	nd***	nd
Asymptomatic/Indeterminate (A)	50	54 ± 10	68 ± 3	50 ± 4
Mild CCC[#] (B1)	50	51 ± 14	58 ± 11	45 ± 14
Moderate/Severe CCC (B2-C-D)	62	51 ± 9	38 ± 9	59 ± 16

Chronic Chagas disease Cardiomyopathy
* Percentage of left ventricle ejection fraction
** Diameter of left ventricle (millimeter)
*** not determined

Supplementary Table 1. Anamnesis of patients with Chagas disease. Sex, age, left ventricle ejection fraction (LVEF), left ventricle diameter (LV).
